# Recurrent *DCC* gene losses during bird evolution

**DOI:** 10.1038/srep37569

**Published:** 2017-02-27

**Authors:** François Friocourt, Anne-Gaelle Lafont, Clémence Kress, Bertrand Pain, Marie Manceau, Sylvie Dufour, Alain Chédotal

**Affiliations:** 1 Sorbonne Universités, UPMC Univ Paris 06, INSERM, CNRS, Institut de la Vision, 17 Rue Moreau, 75012 Paris, France; 2 Muséum National d’Histoire Naturelle, Sorbonne Universités, Research Unit BOREA, Biology of Aquatic Organisms and Ecosystems, CNRS 7208, IRD207, UPMC, UCN, Paris, France; 3 Université Lyon 1, INSERM, INRA, Stem Cell and Brain Research Institute, U1208, USC1361, 69500 Bron, France; 4 Center for Interdisciplinary Research in Biology, CNRS UMR 7241, Collège de France, 75005 Paris, France

## Abstract

During development, midline crossing by axons brings into play highly conserved families of receptors and ligands. The interaction between the secreted ligand Netrin-1 and its receptor Deleted in Colorectal Carcinoma (DCC) is thought to control midline attraction of crossing axons. Here, we studied the evolution of this ligand/receptor couple in birds taking advantage of a wealth of newly sequenced genomes. From phylogeny and synteny analyses we can infer that the *DCC* gene has been conserved in most extant bird species, while two independent events have led to its loss in two avian groups, passeriformes and galliformes. These convergent accidental gene loss events are likely related to chromosome Z rearrangement. We show, using whole-mount immunostaining and 3Disco clearing, that in the nervous system of all birds that have a *DCC* gene, DCC protein expression pattern is similar to other vertebrates. Surprisingly, we show that the early developmental pattern of commissural tracts is comparable in all birds, whether or not they have a DCC receptor. Interestingly, only 4 of the 5 genes encoding secreted netrins, the DCC ligands in vertebrates, were found in birds, but Netrin-5 was absent. Together, these results support a remarkable plasticity of commissural axon guidance mechanisms in birds.

Despite more than 600 million years of evolution, the basic components of the bilaterian brain wiring diagram are highly conserved^1^. One of its signature trait is the presence of two categories of projection neurons: some that connect to target cells located on the same, or ipsilateral, side of the nervous system and others that connect on the opposite, or contralateral, side. The latest are called commissural neurons and they are distributed all along the rostro-caudal axis^2^. The position and spatio-temporal developmental sequence of a common set of commissural tracts (anterior commissure, posterior commissure, fasciculus retroflexus, optic nerve among others) are highly similar among vertebrates^3^,^4^. However, some commissural tracts only exist in some taxa, such as the corpus callosum in placental mammals^5^, or the Mauthner cells in lampreys, teleosts and amphibians^6^. It is assumed that the appearance of novel commissural circuits has allowed the acquisition of novel brain functions and behaviours^2^,^7^.

Understanding the mechanisms controlling the development and patterning of commissural circuits has represented a daunting challenge to developmental neurobiologists since the end of the nineteenth century^8^,^9^. Significant progress has only been made during the past thirty years or so through genetic and biochemical screening. The current model favors a rather simple push-pull mechanism whereby cells at the CNS midline, such as the floor plate in vertebrates, secrete proteins that attract commissural axons and facilitate midline crossing and also repellents that force commissural axons to leave the midline^8^,^10^. Netrin-1, the first midline chemoattractant, was simultaneously identified in *C. elegans* and in chick embryo^11^. The vertebrate *DCC* gene (Deleted in Colorectal Carcinoma^12^), and its homologues in *C. elegans*^13^ and *Drosophila*^14^, encode a transmembrane receptor, mediating Netrin-1 attraction. In these species, *DCC* loss-of-function prevents many commissural axons from crossing the midline, thereby supporting DCC pivotal role in midline guidance^14^,^15^. In mice and human, mutations in *DCC* leads to lethality^15^, movement disorders^16^ and cancers^17^. It was proposed that the *DCC* gene is absent from the chicken genome and that its paralogue, NEOGENIN, mediates NETRIN-1 attraction in this species^18^. However, multiple *in vivo* and *in vitro* studies have shown that in chick embryos, the chemotropic activity of NETRIN-1 on spinal cord commissural axons, enteric neural crest cells and oligodendrocyte precursors is blocked by anti-DCC antibodies^19^,^20^. Moreover, the *in ovo* electroporation of dominant negative constructs of DCC or DCC signaling partners in the chick spinal cord significantly perturbs commissural and motor axon guidance^21^,^22^,^23^. This suggests that a *DCC* gene might exist in the chick genome, which is known to be fragmented and to contain at least 30 microchromosomes^24^. Recently, the annotated genomes of 48 bird species were released^25^ which led us to revisit the evolution history of *DCC* and *NETRIN* genes in birds. We have also performed a comparative analysis of the organization and development of commissural circuits in early bird embryos.

## Results

### *DCC* gene is present in most sauropsid genomes

**Figure 1:**
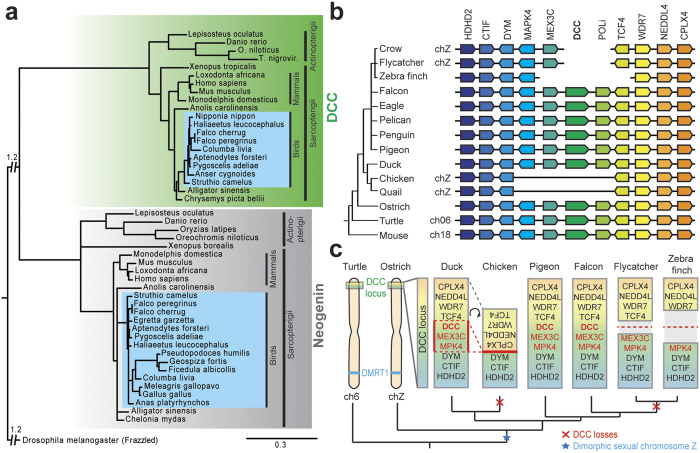
DCC gene has been lost independently twice during bird evolution. (**a**) Consensus phylogenetic tree of vertebrate *DCC* and *NEOGENIN*. Analysis was performed on 47 vertebrate DCC and NEOGENIN amino acid sequences using the Maximum likelihood method, with 1000 bootstrap replicates (for sequence references, see Supplementary Table S1). The tree was rooted using *Drosophila* FRAZZLED sequence as outgroup and branches displaying bootstrap values below 50 were collapsed. For better visualization, we cut out primary branches of a length equivalent to 1.2. (**b**) Conserved genomic synteny of amniotes *DCC* chromosomal region. The Figure displays simplified genomic synteny map comparing positions of *DCC* and its neighboring genes in different amniotes species (For full analysis, see Supplementary Figure S1). Orthologues of each gene are represented in the same color and displayed in the same column. (**c**) Proposed scenario for *DCC* gene loss in the avian lineage. DCC gene loss has occurred twice, independently, in galliformes and in passeriformes.

We first investigated whether a *DCC* gene is present in all available sauropsid genomes. Using NCBI and *Ensembl* genome databases, we found genes annotated as “*DCC*” in several avian, crocodilian and chelonian genomes (see Supplementary Table S1). We performed a phylogenetic analysis to rule out the possibility that the sauropsid *DCC* genes would be in fact Neogenin genes. We reconstructed vertebrate phylogeny of DCC and NEOGENIN proteins, using 47 sequences from 27 amniotes genomes, with drosophila Frazzled receptor (the DCC orthologue in flies) as outgroup (Fig. 1a). In this tree, DCC and NEOGENIN sequences cluster in two different well-supported clades, confirming that the two receptors are encoded by two distinct genes. Sauropsid DCC sequences are encompassed in vertebrate DCC group and recapitulate known phylogeny. Moreover, short branch lengths demonstrate that this gene is highly conserved among all vertebrates. Importantly, Neogenin genes could be found in all bird species investigated. The longer branch for neogenin sequences from passerifomes (Pseudopodoces humilis, Geospiza fortis, Fiducela albicolis) reveals a specific sequence divergence in this group. Together, these results confirm that a *DCC* gene is present in sauropsid including many bird species.

### DCC gene was lost twice in aves evolution

In birds, *DCC* gene is present in most birds major groups^26^ including paleognathes, anseriformes, strisores, columbaves, gruiformes, aequorlitornithes, accipitriformes, coraciimorphes, falconiformes and psittaciformes (Supplementary Table S1). However, in agreement with a previous report^18^, we could not find any *DCC* gene in the presently available chicken *Gallus gallus* genome. This was also the case for two other galliformes: the turkey *Meleagris gallopovo* and the quail *Coturnix japonicus,* suggesting that *DCC* gene is absent from this group. Another striking result was the absence of *DCC* gene in a second major avian group, the passeriformes. Indeed we did not detect *DCC* sequences in any of the eleven passeriformes genomes available on NCBI including the zebra finch *Taenopygia guttata*. For both galliformes and passeriformes we identified *DCC* genes in their respective sister group, namely anseriformes and psittaciformes, which supports independent gene losses (Supplementary Table S1).

To understand the evolutionary history of the *DCC* gene in birds, we investigated the *DCC* genomic region. We performed a physical co-localization of genetic loci on the same chromosome within an individual or species analysis (or synteny) of this particular region in amniotes genomes, i.e. mammals (human, mouse, platypus), testudinian (painted turtle), crocodilian (alligator) and various birds including paleognathes (ostrich), galloanseres (chicken, turkey, duck, goose) and 15 neoaves (Fig. 1b, Supplementary Fig. S1 and Supplementary Table S3-4-5). When present, *DCC* is always located on the sexual chromosome Z in birds, in contrast with other sauropsids and mammals in which *DCC* is on autosomes^27^,^28^.

Synteny analysis showed that this region was highly conserved in amniotes and only few gene rearrangements could be observed between sauropsids and mammals, except from two major gene block losses in galliformes and passeriformes (Fig. 1b and Supplementary Fig. S1).

In galliformes, a block of 12 genes, including *DCC,* was absent from the syntenic region: *MAPK4, ME2, ELAC1, SMAD4, MEX3C, DCC, MBD2, POLI, STARD6, DYNAP, RAB27B*, and *CCDC68* (Supplementary Fig. S1). In addition, a 13th gene, *TCF4* appeared to be missing in turkey (Supplementary Fig. S1). Only two of these genes could be detected elsewhere in these galliformes genomes using tblastn algorithm from NCBI: *MBD2* gene in chicken and quail and *CCDC68* in turkey (Supplementary Fig. S1), but their position in the genome is still undetermined. Moreover, many chromosomal rearrangements could be observed downstream of this region in galliformes, in contrast with the stability of this genomic region in the sister group, anseriformes. In particular, the block of genes nearby this deletion (from *TCF4* to *CPLX4*) is reversed compared to the ancestral sequence. Furthermore, *TCF4* gene, that is located at the border of the deletion block, is annotated at 875 bp from the beginning of Z chromosome (Gallus_gallus-4.0; Ch.Z NC_006127.3). Together this suggests that the *DCC* block was lost in galliformes during a Z chromosome extremity flip as illustrated in Fig. 1c.

In passeriformes, a block of 7 genes, including *DCC*, is also absent from the locus: *DCC, MBD2, POLI, STARD6, DYNAP, RAB27B*, and *CCDC68*. In addition, an 8th gene, *TCF4*, is missing in the White-throated sparrow and Zebra finch available genomes (Supplementary Fig. S1). As for galliformes, none of these genes could be detected using tblastn algorithm on passerifomes genome sequences. This portion of passeriforme genomes is poorly assembled, and it is impossible to determine how these scaffolds are positioned on Z chromosome. Previous studies demonstrated that the Z chromosome has undergone major gene loss and shortening during bird evolution^29^. Our observations are compatible with a scenario of two independent Z chromosome rearrangements in galliformes and passeriformes leading to large chromosomic region losses, including the *DCC* gene block (Fig. 1c).

### Netrin genes, except *NETRIN-5*, are present in all birds

We next investigated whether genes encoding DCC main ligands, the secreted NETRINs^30^,^31^,^32^, were present in known bird genomes. In vertebrates, 5 secreted NETRIN proteins have been described (NETRIN-1 to 5) and are all thought to bind to DCC^12^,^33^,^34^,^35^. Previous study indicates that *NETRIN* genes originated prior to vertebrate radiation^36^. To investigate the evolutionary history of *NETRIN* genes in birds, we reconstructed their phylogeny using amphioxus NETRIN-1 and NETRIN-4 as outgroups (Supplementary Fig. S2). This phylogenetic tree revealed that NETRIN-2 and NETRIN-3 sequences cluster in a single well-supported clade (see Supplementary Fig. S2), demonstrating that these are actually two annotations of the same gene. This observation was confirmed by synteny analysis (data not shown). No particular divergence could be observed for birds NETRIN-1, NETRIN-2/3, or *NETRIN*-*4* sequences, as compared to the other vertebrates. In particular, these genes were also present and conserved in both galliformes and passeriformes (Supplementary Fig. S2). In contrast, long branches suggested that NETRIN-5 sequences are highly divergent among all vertebrates. Furthermore, it was impossible to find any *NETRIN-5* gene in bird genomes (Supplementary Fig. S2). Taken together, these data show that there is no ligand modification counterpart of *DCC* gene loss in galliformes and passeriformes.

### DCC mRNA and protein are not detectable in galliformes and passeriformes

**Figure 2:**
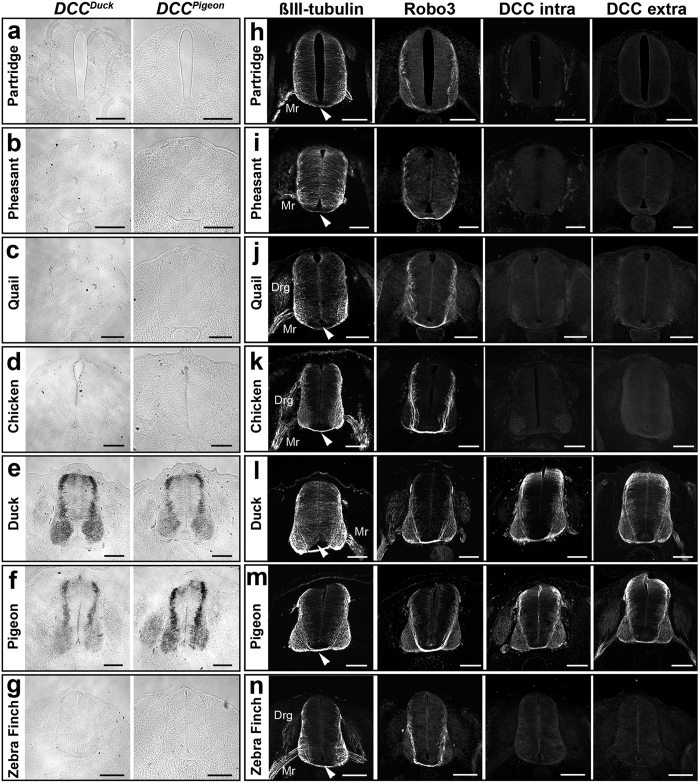
Both DCC mRNA and protein are expressed in bird spinal cord except in galliformes and passeriformes. Spinal cord sections from different birds are stained: partridge (**a,h**), pheasant (**b,i**), quail (**c,j**), chick (**d,k**), duck (**e,l**), pigeon (**f,m**) and zebra finch (**g,n**). (**a–g**) *In situ* hybridization using *DCC* antisense riboprobes cloned from duck and pigeon detect strong expression of DCC mRNA expression in the spinal cord (**e,f**). Duck and pigeon riboprobes cross-react between the two species, but fail to detect *DCC* mRNA in galliformes (**a–d**) and passeriformes (**g**). (**h–n**) Expression of ßIII-Tubulin and ROBO3 was detected in ventral commissures of all spinal cords. Anti-DCC antibodies against the intracellular and extracellular domains detect strong expression of DCC protein in ventral commissural neurons in duck (**l**) and pigeon (**m**) but fail to detect any DCC expression in galliformes (**h–k**) and passeriformes (**n**). Arrows indicate the ventral midline. Abbreviations, Drg, dorsal root ganglia; Mr, motor nerve root. Scale bars: 50 μm.

To confirm the loss of *DCC* in galliformes and passeriformes compared to other bird species, we assessed its mRNA and protein product by performing *in situ* hybridization and immunostaining. We used two riboprobes, cloned respectively from duck and pigeon cDNA, and two different antibodies, specific for DCC extracellular and intracellular domain (see Methods). We performed experiments on different bird embryos from species with available genomic data: chicken (*Gallus gallus*), quail (*Cortunix japonica*), duck (*Anas platyrhynchos*), pigeon (*Columba livia*), and zebra finch (*Taeniopygia guttata*). In addition we used three other galliformes (pheasant, partridge, and quail) assuming that they do not have a *DCC* gene. Pigeon and duck DCC antisense riboprobes could detect *DCC* mRNA in their respective species, while sens riboprobes could not (Fig. S3). In addition, the two probes could detect DCC mRNA in both species (Fig. 2e,f). In contrast, no signal was detectable with either probe in galliformes and passeriformes (Fig. 2a–d,g), strongly suggesting an absence of DCC mRNA in these embryos. Similarly, the two anti-DCC antibodies labeled spinal cord commissures in duck and pigeon embryos but not in embryos from galliformes and passeriformes (Fig. 2l and n). Commissural axons were also immunoreactive for DCC in the hindbrain of ostrich embryos (data not shown). This result confirms genomic data of specific *DCC* gene losses in passeriformes and galliformes. Immunostaining with an antibody against the pan-neuronal marker ßIII-Tubulin showed that the overall organization of axonal tracts was highly similar in spinal cord section from the seven species tested (Fig. 2). Importantly, the ventral spinal cord commissure was present in all birds. We also performed immunostaining with antibodies against the Roundabout 3 (ROBO3) receptor, known to be expressed by growing commissural axons in the spinal cord and hindbrain in developing mammals^37^,^38^, zebrafish^39^ and chicken^40^. A single *ROBO3* gene was detected in all birds (data not shown) and accordingly, ROBO3-immunopositive commissural axons were observed on spinal cord sections from all bird embryos tested (Fig. 2). Thus absence of DCC receptor does not appears to impact bird spinal cord commissural formation.

**Figure 3:**
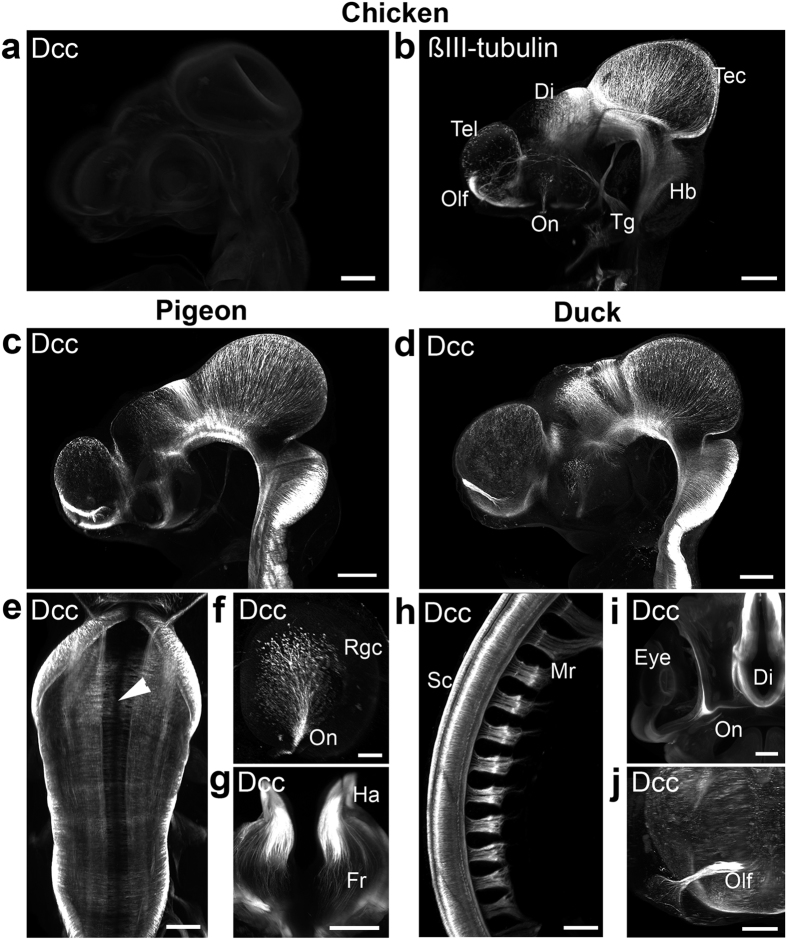
3DISCO analysis of DCC receptor expression in chicken, pigeon and duck embryos. DCC (**a**) and ßIII-Tubulin (**b**) whole-mount immunostaining on HH22 chicken embryos after 3DISCO clearing. No DCC expression is detected in chicken embryos whereas many axonal tracts are strongly labeled with anti-ßIII-Tubulin. (**c–j),** By contrast, DCC is strongly expressed in pigeon (**c,e–g**) and duck (**d,h–j**) embryos at stages equivalent to HH22 or HH28. (**h–j**) in pigeon embryo, DCC is found in commissural axons crossing the floor plate (arrowhead in **e**), in retinal ganglion cells (Rgc) of the retina and the optic nerve (On; **f**), in the habenula nucleus (Ha) and fasciculus retroflexus (Fr; **g**). (**h–j**) in duck embryo, DCC is found in spinal cord (Sc) commissural axons, motor nerve roots (Mr), Rgc in the eye (**i**), optic nerve (On) diencephalon (Di) and olfactory nerve (Olf). Scale bars are 200 μm except (**e,j**), 100 μm and f, 50 μm. Abbreviations, Di: diencephalon; Hb: hindbrain; Tec, tectum; Olf: olfactory nerve; Tel, telencephalon; Tg, trigeminal ganglion.

To further study DCC expression pattern in birds, we performed whole-mount anti-DCC immunostaining, 3DISCO clearing and 3D imaging with light sheet microscopy on chick, pheasant, duck, pigeon and zebra finch embryos at HH21-22. In the chick, early axonal tracts^41^ and peripheral nerves could be labeled with anti-ßIII Tubulin immunostaining but none expressed DCC (Fig. 3a,b and Supplementary Movie S1). We also failed to detect any DCC expression in pheasant and zebra finch embryos (Supplementary Fig. S4), confirming what was found in the spinal cord. In contrast, many axons were immunoreactive for DCC in duck and pigeon embryos (Fig. 3c,d). As previously shown in rodents and xenopus, DCC was broadly expressed by commissural axons in the mesencephalon, diencephalon and rhombencephalon. DCC was also detected in retinal ganglion cells, in the olfactory nerves, motor axons and fasciculus retroflexus (Fig. 3e–j and Supplementary Movie S1). Together, these data confirm that DCC was selectively lost in the galliforme and passeriforme lineages despite its highly conserved expression pattern in other tetrapods.

### Homogenous organization of early commissural tracts in bird embryos

**Figure 4:**
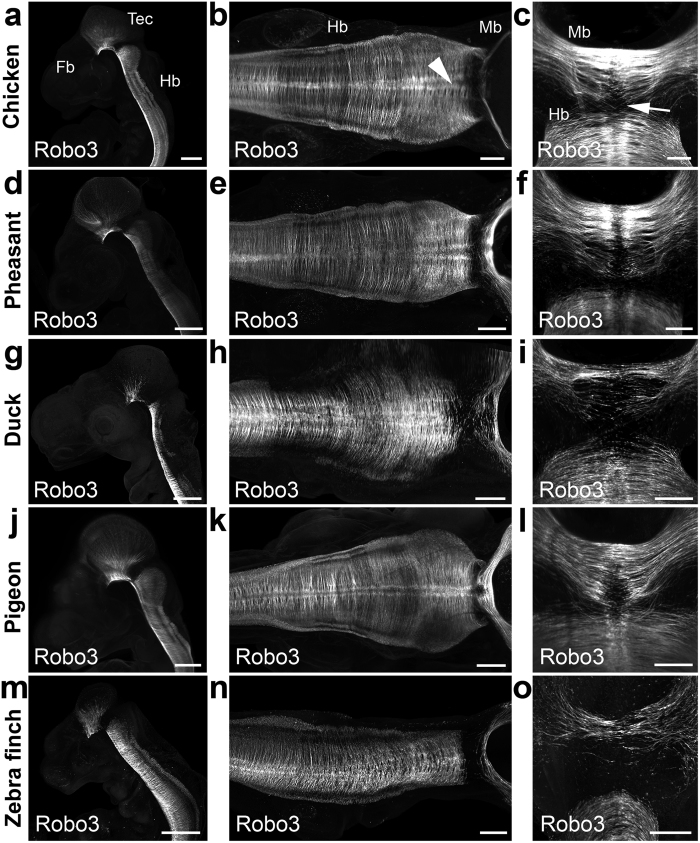
Commissural projections labeled with ROBO3 appear similar in birds with or without DCC. (**a–o**) 3D light sheet images of whole-mount bird embryos labeled with anti-Robo3 antibodies. (**a–c**) in H21-22 chick embryos, Robo3 is expressed by commissural axons in the tectum (Tec), ventral midbrain (Mb) and hindbrain (Hb) but not in the telencephalon (Te). The floor plate is indicated by an arrowhead in (**b**). Commissural axon growth cones (arrow) are seen approaching the midline in (**c**). At equivalent developmental stages, the spatial pattern of Robo3+ commissural projections is similar in pheasant (**d–f**), duck (**g–i**), pigeon (**j–l**) and zebra finch (**m–o**) embryos. Scale bars, 300 μm in (**a**,**d**,**g**,**j**,**m**); 150 μm, in (**b**,**e**,**h**,**k**,**n)**; 100 μm in (**c**,**f**,**i**,**l**,**o**).

To determine if the lack of DCC in passerifomes and galliformes might have resulted in a different organization of their commissural projections, we compared the early development of commissural tracts in the chick with that of pigeon and duck embryos. We used anti-ROBO3 immunostaining to specifically label all posterior commissural tracts and reconstruct their 3D organization. In addition, we used anti-ßIII Tubulin to investigate rostral commissural tracts development. In all embryos, the position, developmental sequence and density of commissural axons were comparable (Fig. 4). As expected from previous work in the mouse, when present, DCC homogeneously stained all spinal cord and hindbrain commissural axons, as does ROBO3 (Fig. 4, Supplementary Fig. S2 and Supplementary Movies S2-S3). The fasciculus retroflexus was also labeled, and no major differences were observed (data not shown). At more rostral levels where ROBO3 was not expressed, commissural tracts such as the posterior commissure or the post optic commissure tract (Supplementary Fig. S4) could be observed with anti-ßIII Tubulin, and no noticeable difference was detected between chick and duck or pigeon embryos.

## Discussion

We found *DCC* genes in representative species of saurians, chelonians, crocodilians, as well as in many bird species, including paleognathes, anseriformes and numerous neoaves. In contrast, *DCC* gene is missing in chicken, in agreement with previous observations^18^, as well as in other galliformes, the turkey and the quail. Furthermore, *DCC* is also absent in passeriformes, such as crow, fly catcher and zebra finch. *DCC* gene was also not found in some bird genomes outside from passerifomes and galliformes (Table S1), however these bird genomes belong to low-coverage genome sequencing groups^42^. Moreover, in these cases, other birds from the same groups have a *DCC* gene, suggesting that its absence is more likely due to incomplete genome sequences, than to a real absence of *DCC* in these species. Phylogeny analysis clearly clustered bird DCC sequences with the DCC of the other sauropsids, and together with mammalian and actinoterpygian sequences in a single DCC clade. By contrast, *NEOGENIN,* the *DCC* paralogue, appears to exist in all birds.

This supports that a *DCC* gene was present in bird ancestor, conserved in various avian groups, but lost in two distinct groups, galliformes and passeriformes. According to current bird phylogeny, these two groups are not closely related^26^,^43^, suggesting that two independent events of *DCC* loss occurred during bird radiation. One is aware that the absence of some genes in current bird genomes could be related to incomplete sequencing of some genomic regions^44^. We show here that the presence or absence of *DCC* mRNA and DCC protein in the brain matched with the presence or absence of *DCC* gene in the corresponding bird genomes. In the phylogeny tree, the short lengths of bird DCC branches indicated that bird DCC sequences did not diverge as compared to the other amniote sequences. In addition DCC expression pattern when present is similar to what has been described in the mouse^14^,^45^. This indicates that the loss of *DCC* in some bird groups was not preceded by any major change of DCC structure and function in the bird lineage. Moreover, synteny analyses showed that both in galliformes and passeriformes, many genes are lost together with *DCC*. This shows that the loss of *DCC* in these birds is not targeted specifically on *DCC* gene, but concerns a whole genomic region. This differs from other recently reported gene loss in birds, such as the loss of *KISS* gene^46^. In this case, a degenerated sequence of KISS could be observed in some bird species, such as duck, zebra finch, and rock pigeon, and a specific loss of this gene in many other birds, including falcon and chicken, suggesting that accumulation of mutations and alteration of function preceded the loss of the gene^46^. Here, the lack of *DCC* would result from independent accidental loss events in both galliformes and passeriformes during Z chromosome recombination. This result has been confirmed in an independent study by Patthey *et al.*^47^

In vertebrates, DCC binds to NETRIN-1^12^,^48^, NETRIN2/3^34^, NETRIN-4^33^ and possibly NETRIN-5^35^. Our phylogeny analyses showed that all birds possess NETRIN-1, 2/3, and 4, as the other osteichthyans with no special divergence. Interestingly, we could not retrieve any *NETRIN-5* sequence in birds, while this gene is present in other amniotes, as well as in teleost fish. This suggests an early loss of *NETRIN-5* in the bird lineage. In mammals, NETRIN-5 is expressed in the developing brain, notably in neurogenic regions^32^ but its function is still largely unknown. Besides the loss of *DCC* in galliformes and passeriformes, the lack of *NETRIN-5* in extant avian species represents another striking specificity of the DCC/NETRIN system in birds.

In the mouse, DCC has been reported to be essential for NETRIN-1 mediated attraction in many different systems^15^ and *DCC* knockouts are not viable^14^. Previous *in vitro* studies have shown that in the chick as well, NETRIN-1 attracts sensory and motor neurons, spinal cord and hindbrain interneurons, GnRH neurons, enteric neural crest cells and oligodendrocyte precursors^20^,^21^,^23^,^49^,^50^,^51^,^52^,^53^. However, the conclusion of several previously published studies deserve to be reconsidered in the light of our results. For instance, the specificity of anti-DCC antibodies used to block NETRIN-1 activity on chick cells^19^,^20^ is highly questionable as there is no DCC in chick genome. Likewise, the axon guidance defects observed after electroporation of truncated or mutated human DCC receptors, or of a ROBO1 ectodomain in chick spinal cord cannot be attributed to a dominant negative effect such as dimerization with endogenous DCC^22^,^23^. Other models, not requiring DCC, should be proposed to explain these results. For instance, the exogenous DCC receptors might bind to and perturb the function of Robo1/Robo2/Robo3 receptors, which are all expressed by chick commissural axons^54^,^55^ and are known DCC partners in mouse neurons^56^,^57^. They might also trap NETRIN-1 thereby titrating it from its other receptors.

Our analysis of early bird embryos failed to reveal any major differences in the development of commissural tracts, regardless of the presence of DCC. Although such differences in commissural systems might exist at later developmental stages, the puzzling question remains on how some bird species coped with the accidental losses of *DCC* and in particular on the identity of the receptor(s) mediating NETRIN-1 chemoattractive activity in these species.

A first obvious candidate is the *DCC* paralogue NEOGENIN, as previously proposed^18^. In the mouse, DCC and NEOGENIN cooperate to attract spinal cord commissural axons to the floor plate, and NEOGENIN can partially compensate for DCC absence in rodents^58^. Phylogeny analysis reveals a divergence in NEOGENIN sequences in passeriformes, which suggests a neofunctionalization of NEOGENIN in this group. As this bird group has lost DCC, we may raise the hypothesis that this neofunctionalization could possibly be related to NEOGENIN taking on DCC function. However, no such NEOGENIN sequence divergence was observed in galliformes, which have also lost DCC. Therefore such a scenario of NEOGENIN neofunctionalization could not apply in this later group. Although NEOGENIN is present in at least some chick spinal cord commissural neurons^18^, it has not yet been shown to be expressed by all NETRIN-1 responsive neurons in this species, and unlike DCC, NEOGENIN has other ligands^59^.

In chick embryo, NETRIN-1 was also shown to bind Down syndrome cell-adhesion molecule (DSCAM) and silencing DSCAM expression in chick spinal cord neurons impairs NETRIN-1 attraction^60^. In rodents, recent *in vivo* studies using knockout mice have challenged this model and suggest that DSCAM^61^ is dispensable for NETRIN-1 attraction of commissural axons. It remains to confirm that this is also the case in passeriformes and galliformes.

Interestingly, DCC was also shown to bind dorsal repulsive axon guidance protein (DRAXIN) a secreted molecule which repels various classes of commissural axons^62^. DRAXIN and NETRIN-1 bind each other and compete for DCC binding^63^. How DRAXIN, which was first isolated in chick embryo^62^, might function in birds that have no DCC is also a mystery, but NEOGENIN could also be involved. Importantly, preliminary analysis of bird genomes indicates that, DSCAM, UNC5A-D and DRAXIN genes exist in all birds (data not shown).

In mammals, DCC plays a role in commissural axon guidance but also in the migration of neural crest cells and of GnRH neurons from the olfactory epithelium^20^,^64^. DCC influences the development of the autonomic innervation of arteries^65^. Moreover, DCC is a dependence receptor that can induce apoptosis in absence of NETRIN-1^66^, a mechanism that might explain its anti-tumorigenic properties^17^. Therefore, one would expect DCC loss to have important consequences on the development or function of many organs in the corresponding bird species, if not fully compensated by other receptors.

Possible loss of SMAD4, also located in the *DCC* genomic region, is particularly interesting as SMAD4 knockout mice are embryonic lethal^67^. However, we could find a SMAD4-like gene on chicken chromosome 25. This gene is conserved and its locus is unchanged in all birds and many vertebrates except mammals, and has been already characterized in xenopus^68^. It will be important to understand how some birds can cope with the absence of SMAD4, or if SMAD4-like could replace SMAD4.

Importantly, cancer has been described in all vertebrates including birds and most of the missing genes such as SMAD4^67^, SKA1^69^, MEX3C^70^ and DCC have been linked to tumorigenesis. Interestingly, there is in chick a high incidence of spontaneous ovarian cancer with a prevalence reaching up to 35% after 3.5 years of age^71^,^72^. This correlates well with the downregulation of DCC expression described in human ovarian tumors^73^,^74^.

In conclusion, our results suggest that commissural axon guidance mechanisms are not conserved between bird species but that overall this does not seem to have a major impact on brain patterning. This illustrates the great plasticity of axon guidance mechanism, and how diverse this system can be among vertebrates. Another example of this diversity was recently reported in mammals, where mutations of few amino acids in mammalian ROBO3 receptor have completely modified its mechanism of action in commissural neurons^56^. To fully appreciate this diversity, it will be essential to reconstruct the phylogenic history of commissural guidance receptors and ligands in vertebrates.

## Methods

### Genomic databases analysis

Protein sequences from annotated genes were extracted from Ensembl or NCBI genome browsers (http://www.ensembl.org/index.html and http://www.ncbi.nlm.nih.gov/nuccore/). The TBLASTN algorithm of the NCBI website (http://blast.ncbi.nlm.nih.gov/Blast.cgi) was used on the genomic databases available when genes where not previously annotated. See Supplementary Table S1 and S2 for complete genome and sequences information.

### Phylogenetic analysis

#### DCC-NEOGENIN Analysis

47 sequences composed of predicted mature netrin-receptor (DCC-NEOGENIN) with N-terminal signal peptide were first aligned using ClustalW^75^, then manually adjusted. The JTT (Jones, Taylor and Thornton) protein substitution matrix of the resulting alignment was determined using ProTest software^76^. Phylogenetic analysis of the NEOGENIN-DCC receptors alignment was performed using the Maximum Likelihood method with 1,000 bootstrap replicates (RaxML software, https://www.phylo.org/portal2). DCC-NEOGENIN homologous *Drosophila melanogaster* FRAZZLED receptor was used as outgroup.

#### NETRIN-1/2/3/5 and NETRIN-4 Analysis

52 sequences for NETRIN-1/2/3/5 and 19 sequences for NETRIN-4, each one composed of a predicted mature NETRIN protein with N-terminal signal peptide, were aligned using ClustalW. The JTT protein substitution matrix of the resulting alignment was determined using ProTest software. Phylogenetic analysis of the NETRIN sequences alignment was performed using the Maximum Likelihood method with 1,000 bootstrap replicates (RaxML software) using *Branchiostoma floridae* NETRIN-1 and NETRIN-4 as outgroups, respectively.

### DCC synteny analysis

Synteny maps of the *DCC* conserved genomic region were reconstructed for mammals (human, mouse, platypus), chelonian (painted turtle), crocodilians (alligator) and birds: paleognathae (ostrich), galloansers (duck, goose, chicken, turkey), and neoaves (falcon, bald eagle, royal eagle, adeli penguin, emperor penguin, pigeon, ibis, egret, cuckoo, chimney swift, hoatzin, zebra finch, sparrow, flycatcher and crow). Analyses of *DCC* neighbouring genes were performed manually using complete or preliminary annotated genome sequences from NCBI genome browser (http://www.ncbi.nlm.nih.gov/gene/), including numerous unplaced genomic scaffolds (see Supplementary Table S3 for references and locations of the genes used in the synteny analysis, Table S4 for a complete list of the genes used in this analysis, and Table S5 for a complete list of species used in the analysis). To complete this analysis we used TBLASTN algorithm on NCBI database to identify non-annotated DCC neighbouring genes and confirm gene absence (http://blast.ncbi.nlm.nih.gov/Blast.cgi).

### Animal sampling

Eggs from chicken *Gallus gallus*, duck *Anas platyrhynchos*, zebra finch *Taenopygia guttata*, pigeon *Columba livia*, quail *Coturnix japonica*, pheasant *Phasianus colchicus* and partridge *Perdrix perdrix* were incubated at 37 °C in humid conditions. Embryos were collected at different time points depending on their embryological stage. All procedures were performed in accordance to the guidelines approved by French Ministry of Agriculture and UPMC University ethic committee. Stage determination was done according to literature^77^,^78^,^79^. Exact number of embryos collected per stage is presented in Supplementary Table S6. Embryos were first transferred to ice-cold PBS 1X; from E8, the nervous system were dissected and all embryos were then fixed by immersion in 4% paraformaldehyde overnight at 4 °C. Samples were transferred to PBS 1X and kept at 4 °C until use.

For whole-mount immunostaining, samples were dehydrated in methanol (MeOH 50%in PBS 1X - MeOH 80% in PBS 1X - MeOH 100%) and incubated overnight in MeOH with 5%H_2_O_2_ to suppress blood auto-fluorescence. Samples were then rehydrated (MeOH 100%- MeOH 80% in PBS 1X - MeOH 50%in PBS 1X - PBS 1X) and kept in PBS 1X at 4 °C until use.

### Histochemistry

#### *In situ* hybridization

Tissue sectioning and *in situ* hybridization were performed as previously described^37^. Pigeon *DCC* probe was designed in highly conserved domain of DCC gene coding from Fibronectin 5 to P1 domain. The sequence was amplified from pigeon embryos cDNA using following primers (Forward: 5′- CAGTAGGTGTCCAGGCTGTTG - 3′; Reverse: 5′- CCCGTTGGCTTCTCCATGTTC - 3′) and cloned into pCRII-TOPO plasmid (ThermoFisher). The Duck DCC cDNA was kindly provided by Dr Sara Wilson.

#### Sections immunostaining

Immunostaining were performed as previously described^37^. The following primary antibodies were used: mouse anti-βIII Tubulin (1:1000, MMS435P-Covance), goat anti-ROBO3 (1:500, AF3076-R&D), goat anti-DCC (1:400, Sc-6535-Santacruz, raised against intracellular C-terminal domain of human DCC), mouse anti-DCC (1:300, AF-OP45-Calbiochem, raised against extracellular domain of human DCC). Corresponding secondary antibodies were used: donkey anti-rabbit Alexa488 (1:500, 711-545-152-Jackson), bovine anti-goat Cy3 (1:500, 805-165-180, Jackson), donkey anti-mouse cy3 (1:500, 715-165-150-Jackson), goat anti-mouse DL649 (1:500, 115-495-205-Jackson). Sections were counterstained with Hoechst and examined with a fluorescent microscope (DM6000, Leica) coupled to a CoolSnapHQ camera (Roper Scientific).

#### Whole-mount Immunostaining

Samples were incubated at room temperature (RT) in a solution (PBSGT) of PBS 1X containing 0.2% gelatin (Prolabo), 0.5% Triton X-100 (Sigma-Aldrich) and 0.01% thimerosal (Sigma-Aldrich) for 3 h (E4-E6) or 8 h. Samples were next transferred to PBSGT containing the primary antibodies and placed at 37 °C, with rotation at 70 rpm, for 4 days (E4-E6) or 7 days. Primary antibodies used were the following: mouse anti-βIII Tubulin (1:1000, MMS435P-Covance), goat anti-ROBO3 (1:400, AF3076 R&D), goat anti-DCC (1:400, Sc-6535 Santacruz) and mouse anti-DCC (1:300, AF-OP45-Calbiochem). Samples were then washed 3 times in PBSGT for 2 h at RT and incubated for 24 h at 37 °C in secondary antibody diluted in PBSGT. Secondary antibodies used were the following: donkey anti-rabbit Alexa647 (1:500, 711-605-152-Jackson), bovine anti-goat Cy3 (1:500, 805-165-180, Jackson) and donkey anti-mouse Alexa488 (1:500, A21202-Lifetechnolgie). After 4 washes of 2 h in PBSGT at RT, samples were stored at 4 °C in PBS until clearing.

Small samples (E4) were included in agarose 1.5% prior tissue clearing for better positioning in the ultramicroscope chamber.

Tissue clearing was performed using 3DISCO-clearing procedure as previously described^80^. Samples are stored in dibenzylether (DBE) in light protected glass vials at RT.

#### Ultramicroscopy

3D imaging was performed with an ultramicroscope (LaVision BioTec) using ImspectorPro software (LaVision BioTec). The light sheet was generated by a laser (wavelength 488 and 561 nm, Coherent Sapphire Laser and 640 nm, Coherent OBIS 640–100LX laser, LaVision BioTec) and two cylindrical lenses. A binocular stereomicroscope (MXV10, Olympus) with a 2X objective (MVPLAPO, Olympus) was used at different magnifications (1.25×, 1.6×, 2×, 2.5×, 3.2× and 4×). Samples were placed in an imaging reservoir made of 100% quartz (LaVision BioTec) filled with DBE and illuminated from the side by the laser light. Images were acquired with a PCO Edge SCMOS CCD camera (LaVision BioTec).

Image processing was performed using Imaris software (Bitmap), as described previously^80^.

## Additional Information

**How to cite this article**: Friocourt, F. *et al*. Recurrent *DCC* gene losses during bird evolution. *Sci. Rep.* **7**, 37569; doi: 10.1038/srep37569 (2017).

**Publisher’s note:** Springer Nature remains neutral with regard to jurisdictional claims in published maps and institutional affiliations.

## Supplementary Material

Supplementary Movie S1

Supplementary Movie S2

Supplementary Movie S3

Supplementary Information

## References

[b1] Wray G. A. Molecular clocks and the early evolution of metazoan nervous systems. Philos. Trans. R. Soc. B Biol. Sci. 370 (2015). 10.1098/rstb.2015.0046 PMC4650124 26554040

[b2] Chédotal A. Development and plasticity of commissural circuits: from locomotion to brain repair. Trends Neurosci. 37, 551–562 (2014). 25220044 10.1016/j.tins.2014.08.009

[b3] Bianco I. H. & Wilson S. W. The habenular nuclei: a conserved asymmetric relay station in the vertebrate brain. Philos. Trans. R. Soc. B Biol. Sci. 364, 1005–1020 (2009). 10.1098/rstb.2008.0213 PMC2666075 19064356

[b4] Figdor M. C. & Stern C. D. Segmental organization of embryonic diencephalon. Nature 363, 630–634 (1993). 8510755 10.1038/363630a0

[b5] Suárez R., Gobius I. & Richards L. J. Evolution and development of interhemispheric connections in the vertebrate forebrain. Front. Hum. Neurosci. 8, 497 (2014). 25071525 10.3389/fnhum.2014.00497 PMC4094842

[b6] Korn H. & Faber D. S. The Mauthner cell half a century later: a neurobiological model for decision-making? Neuron 47, 13–28 (2005). 15996545 10.1016/j.neuron.2005.05.019

[b7] Goulding M. Circuits controlling vertebrate locomotion: moving in a new direction. Nat. Rev. Neurosci. 10, 507–518 (2009). 19543221 10.1038/nrn2608 PMC2847453

[b8] Tessier-Lavigne M. & Goodman C. S. The molecular biology of axon guidance. Science (80-). 274, 1123–1133 (1996). 10.1126/science.274.5290.1123 8895455

[b9] Ramon y Cajal S. La rétine des vertébrés. Cellule 1, 121–247 (1892).

[b10] Chédotal A. Further tales of the midline. Curr. Opin. Neurobiol. 21, 68–75 (2011). 20724139 10.1016/j.conb.2010.07.008

[b11] Serafini T. . The netrins define a family of axon outgrowth-promoting proteins homologous to C. elegans UNC-6. Cell 78, 409–424 (1994). 8062384 10.1016/0092-8674(94)90420-0

[b12] Keino-Masu K. . Deleted in Colorectal Cancer (DCC) encodes a netrin receptor. Cell 87, 175–185 (1996). 8861902 10.1016/s0092-8674(00)81336-7

[b13] Chan S. S. Y. . UNC-40, a C. elegans homolog of DCC (Deleted in Colorectal Cancer), is required in motile cells responding to UNC-6 netrin cues. Cell 87, 187–195 (1996). 8861903 10.1016/s0092-8674(00)81337-9

[b14] Kolodziej P. A. . frazzled encodes a Drosophila member of the DCC immunoglobulin subfamily and is required for CNS and motor axon guidance. Cell 87, 197–204 (1996). 8861904 10.1016/s0092-8674(00)81338-0

[b15] Fazeli A. . Phenotype of mice lacking functional Deleted in colorectal cancer (Dcc) gene. Nature 386, 796–804 (1997). 9126737 10.1038/386796a0

[b16] Srour M. . Mutations in DCC cause congenital mirror movements. Science (80-). 328 (2010). 10.1126/science.1186463 20431009

[b17] Castets M. . DCC constrains tumour progression via its dependence receptor activity. Nature 482, 534–537 (2011). 22158121 10.1038/nature10708

[b18] Phan K. D. . Neogenin may functionally substitute for Dcc in chicken. PLoS One 6, e22072 (2011). 21779375 10.1371/journal.pone.0022072 PMC3133656

[b19] Tsai H.-H., Tessier-Lavigne M. & Miller R. H. Netrin 1 mediates spinal cord oligodendrocyte precursor dispersal. Development 130, 2095–2105 (2003). 12668624 10.1242/dev.00424

[b20] Jiang Y., Liu M. T. & Gershon M. D. Netrins and DCC in the guidance of migrating neural crest-derived cells in the developing bowel and pancreas. Dev. Biol. 258, 364–384 (2003). 12798294 10.1016/s0012-1606(03)00136-2

[b21] Liu G. . Netrin requires focal adhesion kinase and Src family kinases for axon outgrowth and attraction. Nat. Neurosci. 7, 1222–1232 (2004). 15494732 10.1038/nn1331 PMC2266630

[b22] Tcherkezian J., Brittis P. A., Thomas F., Roux P. P. & Flanagan J. G. Transmembrane Receptor DCC Associates with Protein Synthesis Machinery and Regulates Translation. Cell 141, 1–13 (2010). 10.1016/j.cell.2010.04.008 PMC2881594 20434207

[b23] Bai G. . Presenilin-dependent receptor processing is required for axon guidance. Cell 144, 106–118 (2011). 21215373 10.1016/j.cell.2010.11.053 PMC3034090

[b24] Hillier L. W. . Sequencing and comparative analysis of the chicken genome provide unique perspectives on vertebrate evolution. Nature 432, 695 – 716 (2004). 15592404 10.1038/nature03154

[b25] Zhang G. . Comparative genomics reveals insights into avian genome evolution and adaptation. Science (80-). 346, 1311–1320 (2014). 10.1126/science.1251385 PMC4390078 25504712

[b26] Prum R. O. . A comprehensive phylogeny of birds (Aves) using targeted next-generation DNA sequencing. Nature 3–11 doi: 10.1038/nature15697 (2015). 26444237

[b27] Kawagoshi T., Uno Y., Matsubara K., Matsuda Y. & Nishida C. The ZW micro-sex chromosomes of the chinese soft-shelled turtle (pelodiscus sinensis, trionychidae, testudines) have the same origin as chicken chromosome 15. Cytogenet. Genome Res. 125, 125–131 (2009). 19729916 10.1159/000227837

[b28] Kawai A. . Different origins of bird and reptile sex chromosomes inferred from comparative mapping of chicken Z-linked genes. Cytogenet. Genome Res. 117, 92–102 (2007). 17675849 10.1159/000103169

[b29] Zhou Q. . Complex evolutionary trajectories of sex chromosomes across bird taxa. Science (80-). 346, 1246338–1246338 (2014). 10.1126/science.1246338 PMC6445272 25504727

[b30] Kennedy T. E., Serafini T., de la Torre J. & Tessier-Lavigne M. Netrins are diffusible chemotropic factors for commissural axons in the embryonic spinal cord. Cell 78, 425–435 (1994). 8062385 10.1016/0092-8674(94)90421-9

[b31] Lai Wing Sun K., Correia J. P. & Kennedy T. E. Netrins: versatile extracellular cues with diverse functions. Development 138, 2153–2169 (2011). 21558366 10.1242/dev.044529

[b32] Yamagishi S. . Netrin-5 is highly expressed in neurogenic regions of the adult brain. Front. Cell. Neurosci. 9, 1–9 (2015). 25941474 10.3389/fncel.2015.00146 PMC4403520

[b33] Qin S., Yu L., Gao Y., Zhou R. & Zhang C. Characterization of the receptors for axon guidance factor netrin-4 and identification of the binding domains. Mol. Cell. Neurosci. 34, 243–250 (2007). 17174565 10.1016/j.mcn.2006.11.002

[b34] Wang H., Copeland N. G., Gilbert D. J., Jenkins N. a. & Tessier-Lavigne M. Netrin-3, a mouse homolog of human NTN2L, is highly expressed in sensory ganglia and shows differential binding to netrin receptors. J. Neurosci. 19, 4938–4947 (1999). 10366627 10.1523/JNEUROSCI.19-12-04938.1999 PMC6782680

[b35] Garrett A. M. . Analysis of Expression Pattern and Genetic Deletion of Netrin5 in the Developing Mouse. Front. Mol. Neurosci. 9, 1–14 (2016). 26858598 10.3389/fnmol.2016.00003 PMC4726805

[b36] Leclère L. & Rentzsch F. Repeated evolution of identical domain architecture in metazoan netrin domain-containing proteins. Genome Biol. Evol. 4, 883–899 (2012). 22813778 10.1093/gbe/evs061 PMC3516229

[b37] Marillat V. . The slit receptor Rig-1/Robo3 controls midline crossing by hindbrain precerebellar neurons and axons. Neuron 43, 69–79 (2004). 15233918 10.1016/j.neuron.2004.06.018

[b38] Sabatier C. . The divergent Robo family protein rig-1/Robo3 is a negative regulator of slit responsiveness required for midline crossing by commissural axons. Cell 117, 157–169 (2004). 15084255 10.1016/s0092-8674(04)00303-4

[b39] Burgess H. A., Johnson S. L. & Granato M. Unidirectional startle responses and disrupted left-right co-ordination of motor behaviors in robo3 mutant zebrafish. Genes, Brain Behav. 8, 500–511 (2009). 19496826 10.1111/j.1601-183X.2009.00499.x PMC2752477

[b40] Escalante A., Murillo B., Morenilla-Palao C., Klar A. & Herrera E. Zic2-Dependent Axon Midline Avoidance Controls the Formation of Major Ipsilateral Tracts in the CNS. Neuron 80, 1392–1406 (2013). 24360543 10.1016/j.neuron.2013.10.007

[b41] Chédotal A., Pourquié O. & Sotelo C. Initial tract formation in the brain of the chick embryo: selective expression of the BEN/SC1/DM-GRASP cell adhesion molecule. Eur. J. Neurosci. 7, 198–212 (1995). 7757257 10.1111/j.1460-9568.1995.tb01056.x

[b42] Zhang G. . Comparative genomic data of the Avian Phylogenomics Project. 1–8 (2014). 10.1186/2047-217X-3-26 PMC4322804 25671091

[b43] Jarvis E., Mirarab S., Aberer A., Li B. & Houde P. Whole-genome analyses resolve early branches in the tree of life of modern birds. Science (80-.). 346, 1126–1138 (2014). 10.1126/science.1253451 PMC4405904 25504713

[b44] Hron T., Pajer P., Pačes J., Bartůněk P. & Elleder D. Hidden genes in birds. Genome Biol. 16, 164 (2015). 26283656 10.1186/s13059-015-0724-z PMC4539667

[b45] Shu T., Valentino K. M., Seaman C., Cooper H. M. & Richards L. J. Expression of the netrin-1 receptor, deleted in colorectal cancer (DCC), is largely confined to projecting neurons in the developing forebrain. J. Comp. Neurol. 416, 201–212 (2000). 10581466 10.1002/(sici)1096-9861(20000110)416:2<201::aid-cne6>3.0.co;2-z

[b46] Pasquier J. . Looking for the bird Kiss: evolutionary scenario in sauropsids. BMC Evol. Biol. 14, 30 (2014). 24552453 10.1186/1471-2148-14-30 PMC4015844

[b47] Patthey C. . Evolution of the functionally conserved DCC gene in birds. Sci. Rep. 7, 42029 doi: 10.1038/srep42029 (2017). PMC5327406 28240293

[b48] Jain R. a., Bell H., Lim A., Chien C.-B. & Granato M. Mirror Movement-Like Defects in Startle Behavior of Zebrafish dcc Mutants Are Caused by Aberrant Midline Guidance of Identified Descending Hindbrain Neurons. J. Neurosci. 34, 2898–2909 (2014). 24553931 10.1523/JNEUROSCI.2420-13.2014 PMC3931503

[b49] Lemons M. L. . Integrins and cAMP mediate netrin-induced growth cone collapse. Brain Res. 1537, 46–58 (2013). 24001590 10.1016/j.brainres.2013.08.045 PMC3833899

[b50] Murray A., Naeem A., Barnes S. H., Drescher U. & Guthrie S. Slit and Netrin-1 guide cranial motor axon pathfinding via Rho-kinase, myosin light chain kinase and myosin II. Neural Dev. 5, 16 (2010). 20569485 10.1186/1749-8104-5-16 PMC2907369

[b51] Tsai H.-H., Macklin W. B. & Miller R. H. Netrin-1 is required for the normal development of spinal cord oligodendrocytes. J. Neurosci. 26, 1913–1922 (2006). 16481423 10.1523/JNEUROSCI.3571-05.2006 PMC6674920

[b52] Murakami S., Ohki-Hamazaki H., Watanabe K., Lkenaka K. & Ono K. Netrin 1 provides a chemoattractive cue for the ventral migration of GnRH neurons in the chick forebrain. J. Comp. Neurol. 518, 2019–2034 (2010). 20394056 10.1002/cne.22319

[b53] Shoja-Taheri F., DeMarco A. & Mastick G. S. Netrin1-DCC-Mediated Attraction Guides Post-Crossing Commissural Axons in the Hindbrain. J. Neurosci. 35, 11707–11718 (2015). 26290247 10.1523/JNEUROSCI.0613-15.2015 PMC4540804

[b54] Reeber S. L. . Manipulating Robo expression *in vivo* perturbs commissural axon pathfinding in the chick spinal cord. J. Neurosci. 28, 8698–8708 (2008). 18753371 10.1523/JNEUROSCI.1479-08.2008 PMC2886497

[b55] Philipp M. . RabGDI controls axonal midline crossing by regulating Robo1 surface expression. Neural Dev. 7, 36 (2012). 23140504 10.1186/1749-8104-7-36 PMC3520763

[b56] Zelina P. . Signaling Switch of the Axon Guidance Receptor Robo3 during Vertebrate Evolution. Neuron 84, 1–15 (2014). 25433640 10.1016/j.neuron.2014.11.004

[b57] Stein E., Zou Y., Poo M. & Tessier-Lavigne M. Binding of DCC by netrin-1 to mediate axon guidance independent of adenosine A2B receptor activation. Science (80-). 291, 1976–1982 (2001). 10.1126/science.1059391 11239160

[b58] Xu K., Wu Z., Renier N. & Antipenko A. Structures of netrin-1 bound to two receptors provide insight into its axon guidance mechanism. Science (80-). 344, 1275–1279 (2014). 10.1126/science.1255149 PMC4369087 24876346

[b59] Wilson N. H. & Key B. Neogenin: One receptor, many functions. Int. J. Biochem. Cell Biol. 39, 874–878 (2007). 17137827 10.1016/j.biocel.2006.10.023

[b60] Liu G. . DSCAM functions as a netrin receptor in commissural axon pathfinding. Proc. Natl. Acad. Sci. USA 106, 2951–2956 (2009). 19196994 10.1073/pnas.0811083106 PMC2650370

[b61] Palmesino E., Haddick P. C. G., Tessier-Lavigne M. & Kania A. Genetic Analysis of DSCAM’s Role as a Netrin-1 Receptor in Vertebrates. J. Neurosci. 32, 411–416 (2012). 22238077 10.1523/JNEUROSCI.3563-11.2012 PMC6621089

[b62] Islam S. M. . Draxin, a repulsive guidance protein for spinal cord and forebrain commissures. Science (80-). 323, 388–393 (2009). 10.1126/science.1165187 19150847

[b63] Gao X. . A Floor-Plate Extracellular Protein-Protein Interaction Screen Identifies Draxin as a Secreted Netrin-1 Antagonist. Cell Rep. 12, 694–708 (2015). 26190107 10.1016/j.celrep.2015.06.047

[b64] Schwarting G. A., Raitcheva Ã. D., Bless E. P., Ackerman S. L. & Tobet S. Netrin 1-mediated chemoattraction regulates the migratory pathway of LHRH neurons. Eur. J. Neurosci. 19, 11–20 (2004). 14750959 10.1111/j.1460-9568.2004.03094.x

[b65] Brunet I. . Netrin-1 controls sympathetic arterial innervation. J. Clin. Invest. 124, 3230–3240 (2014). 24937433 10.1172/JCI75181 PMC4071369

[b66] Mehlen P. . The DCC gene product induces apoptosis by a mechanism requiring receptor proteolysis. Nature 395, 801–804 (1998). 9796814 10.1038/27441

[b67] Takaku K. . Intestinal tumorigenesis in compound mutant mice of both Dpc4 (Smad4) and Apc genes. Cell 92, 645–656 (1998). 9506519 10.1016/s0092-8674(00)81132-0

[b68] Masuyama N., Hanafusa H., Kusakabe M., Shibuya H. & Nishida E. Identification of Two Smad4 Proteins in Xenopus. J. Biol. Chem. 274, 12163–12170 (1999). 10207044 10.1074/jbc.274.17.12163

[b69] Welburn J. P. I. . The Human Kinetochore Ska1 Complex Facilitates Microtubule Depolymerization-Coupled Motility. Dev. Cell 16, 374–385 (2009). 19289083 10.1016/j.devcel.2009.01.011 PMC2746561

[b70] Burrell R. A. . Replication stress links structural and numerical cancer chromosomal instability. Nature 494, 492–496 (2013). 23446422 10.1038/nature11935 PMC4636055

[b71] Johnson P. a. & Giles J. R. The hen as a model of ovarian cancer. Nat. Rev. Cancer 13, 432–436 (2013). 23676850 10.1038/nrc3535

[b72] Hawkridge A. M. The chicken model of spontaneous ovarian cancer. Proteomics - Clin. Appl. 8, 689–699 (2014). 25130871 10.1002/prca.201300135 PMC4924577

[b73] Meimei L. . Lost expression of DCC gene in ovarian cancer and its inhibition in ovarian cancer cells. Med. Oncol. 28, 282–289 (2011). 20054719 10.1007/s12032-009-9400-z

[b74] Papanastasiou A. D., Pampalakis G., Katsaros D. & Sotiropoulou G. Netrin-1 overexpression is predictive of ovarian malignancies. Oncotarget 2, 363–367 (2011). 21789787 10.18632/oncotarget.258 PMC3248189

[b75] Thompson J. D., Higgins D. G. & Gibson T. J. CLUSTAL W: improving the sensitivity of progressive multiple sequence alignment through sequence weighting, position-specific gap penalties and weight matrix choice. Nucleic Acids Res. 22, 4673–4680 (1994). 7984417 10.1093/nar/22.22.4673 PMC308517

[b76] Abascal F., Zardoya R. & Posada D. ProtTest: Selection of best-fit models of protein evolution. Bioinformatics 21, 2104–2105 (2005). 15647292 10.1093/bioinformatics/bti263

[b77] Hamburger V. & Hamilton H. L. A series of normal stages in the development of the chick embryo. J. Morphol. 88, 49–92 (1951). 24539719

[b78] Ainsworth S. J., Stanley R. L. & Evans D. J. R. Developmental stages of the Japanese quail. J. Anat. 216, 3–15 (2010). 19929907 10.1111/j.1469-7580.2009.01173.x PMC2807971

[b79] Murray J. R., Varian-Ramos C. W., Welch Z. S. & Saha M. S. Embryological staging of the Zebra Finch, *Taeniopygia guttata*. J. Morphol. 274, 1090–1110 (2013). 23813920 10.1002/jmor.20165 PMC4239009

[b80] Belle M. . A Simple Method for 3D Analysis of Immunolabeled Axonal Tracts in a Transparent Nervous System. Cell Rep. 9, 1191–1201 (2014). 25456121 10.1016/j.celrep.2014.10.037

