# Supplementary material for: Recurrent DCC gene losses during bird evolution
Source: Sci Rep. 2017 Feb 27;7:37569. doi: 10.1038/srep37569 (PMC5327424; doi:10.1038/srep37569)
Supplement: Supplementary Information [file srep37569-s4.pdf]

Supplementary materials for

Recurrent *DCC* gene losses during bird evolution

François Friocourt[1], Anne-Gaelle Lafont[2], Clémence Kress[3], Bertrand Pain[3] Marie Manceau[4], Sylvie Dufour[2] and Alain Chédotal[1,*]

1 Sorbonne Universités, UPMC Univ Paris 06, INSERM, CNRS, Institut de la Vision, 17 Rue Moreau, 75012 Paris, France

2Muséum National d'Histoire Naturelle, Sorbonne Universités, Research Unit BOREA, Biology of Aquatic Organisms and Ecosystems, CNRS 7208, IRD207, UPMC, UCN, Paris, France

3Université Lyon 1, INSERM, INRA, Stem Cell and Brain Research Institute, U1208, USC1361, 69500 Bron, France

4Center for Interdisciplinary Research in Biology, CNRS UMR 7241, Collège de France, 75005 Paris, France

* corresponding author

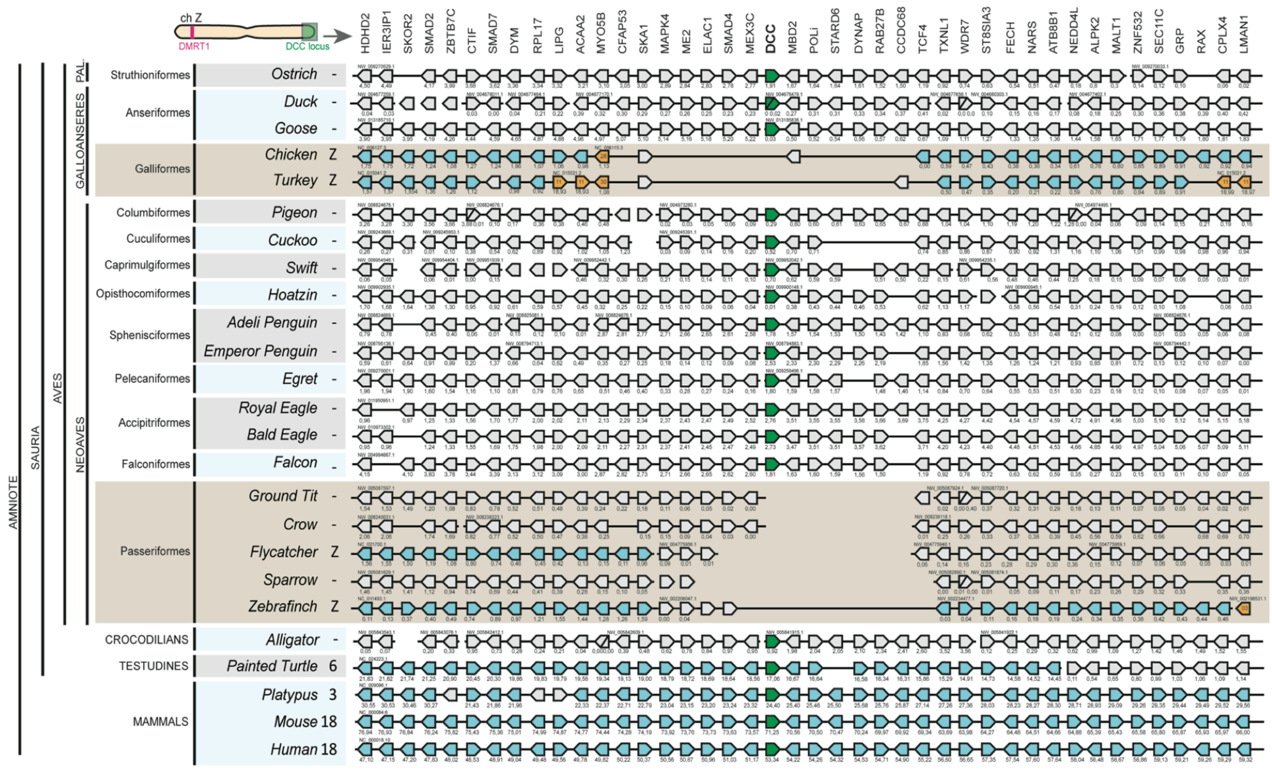

Figure S1 – Synteny analysis of DCC chromosomic region in amniotes.

Genomic synteny map comparing positions of *DCC* and its neighbouring genes in different amniote species, including a wide variety of birds. Orthologues of each gene are displayed in the same column. Genes from unplaced genomic scaffolds are represented in grey. Genes that have been positioned on chromosomes on annotated genomes are represented in blue if they are on the same chromosome as *DCC* or on bird dimorphic sexual Z chromosome, and in orange if they are located on another chromosome. Chromosome or scaffold references are indicated above each independent sequence.

The genes shown in this figure are not necessary presented in the same order as their actual locations on the chromosomes and scaffolds, which are indicated in 10^6 base pairs. A broken line symbolizes the location of a gene on different scaffolds or contigs. The detailed genomic locations of all the genes are given in Supplementary Table S3. Detailed gene names and species names are given in Supplementary Table S4 and S5 respectively.

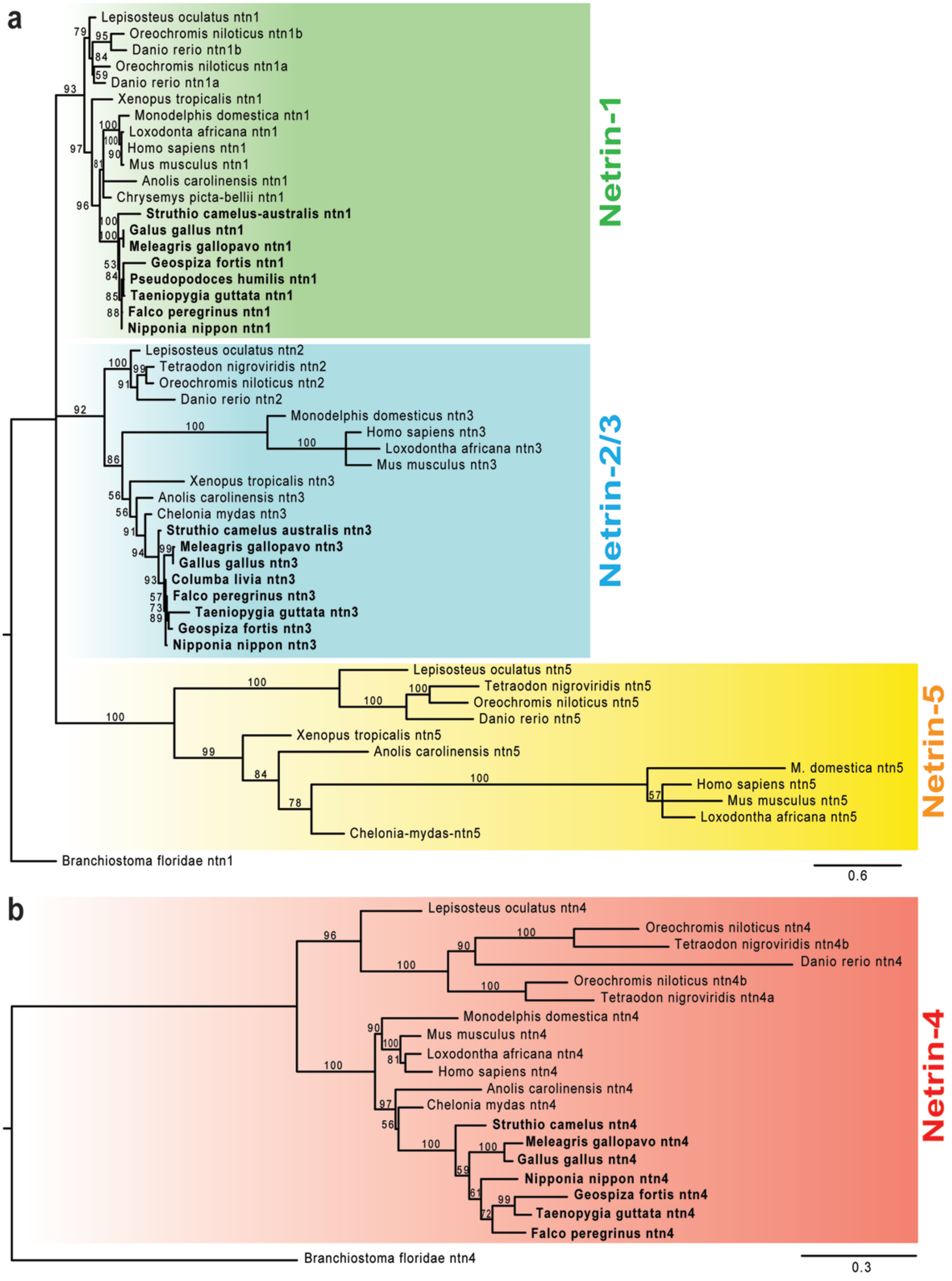

Figure S2 – Consensus phylogenetic tree of vertebrate NETRIN-1/2/3/5 and NETRIN-4. Phylogenetic analysis of 52 vertebrate NETRIN-1, NETRIN-2/3 and NETRIN-5 amino acid sequences (**a**) and 19 vertebrate NETRIN-4 amino acid sequences (**b**) were performed using the Maximum likelihood method, with 1000 bootstrap replicates (for the references of sequences, see Supplementary Table S1). The number displayed at each branch node indicates bootstrap value in percentage. Only values above 50% are indicated. The tree was rooted using *Branchiostoma floridae* NETRIN-1 and NETRIN-4 sequences as outgroups, respectively. Vertebrate NETRIN-1 group is indicated in red, NETRIN-2/3 in green, NETRIN-5 in yellow and NETRIN-4 in red. Bird protein sequences are presented in bold.

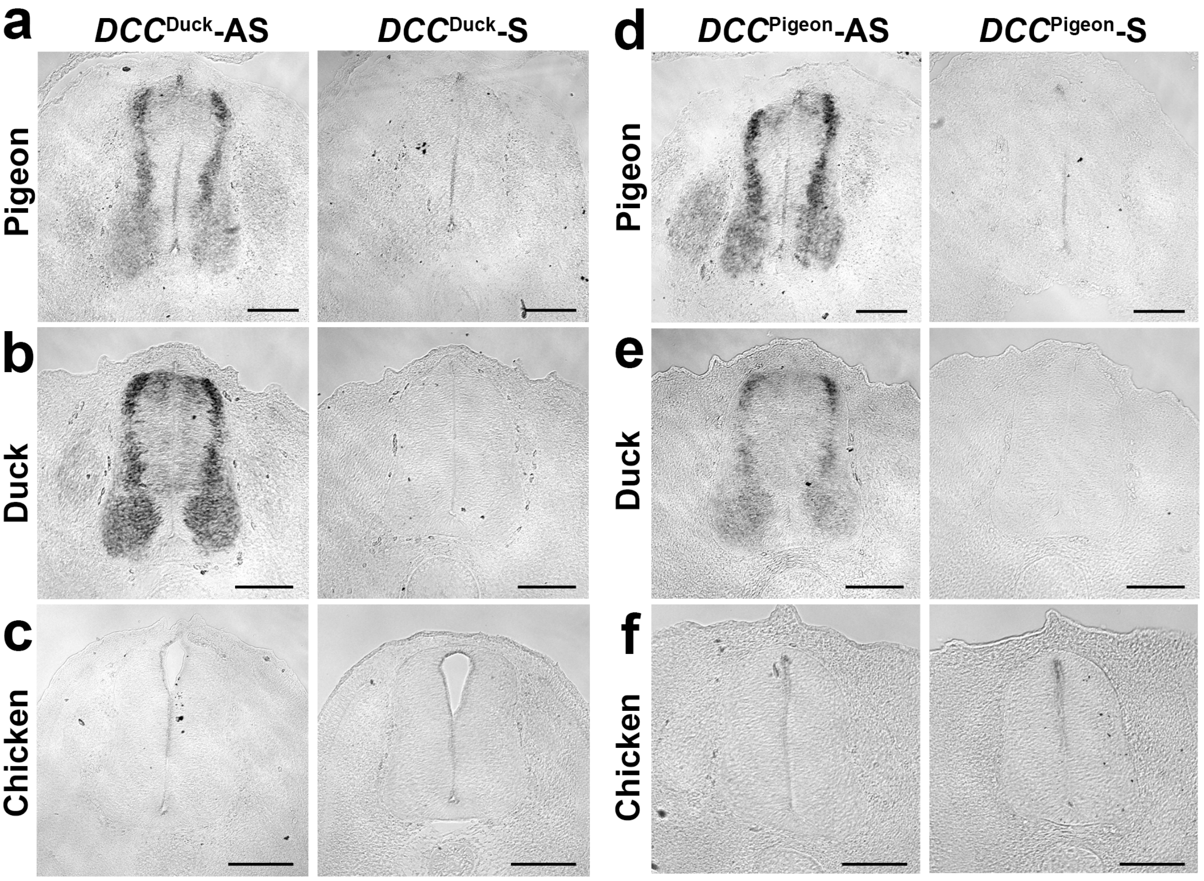

Figure S3. *In situ* hybridization for *DCC* in pigeon, duck and chicken spinal cord. a-c. *In situ* hybridization using *DCC* antisense (AS) riboprobe amplified from duck cDNAs shows labeling in spinal cord interneurons and motor neurons territory from pigeon (**a**), duck (**b**), but not chicken (**c**) embryos. No labeling is seen with sense (S) riboprobes. **d-f.** *In situ* hybridization using *DCC* antisense (AS) riboprobe from pigeon cDNA shows labeling in spinal cord from pigeon (**d**), duck (**e**), but not chicken (**f**) embryos. No staining is seen with sense (S) riboprobes. Scale bars: 50μm.

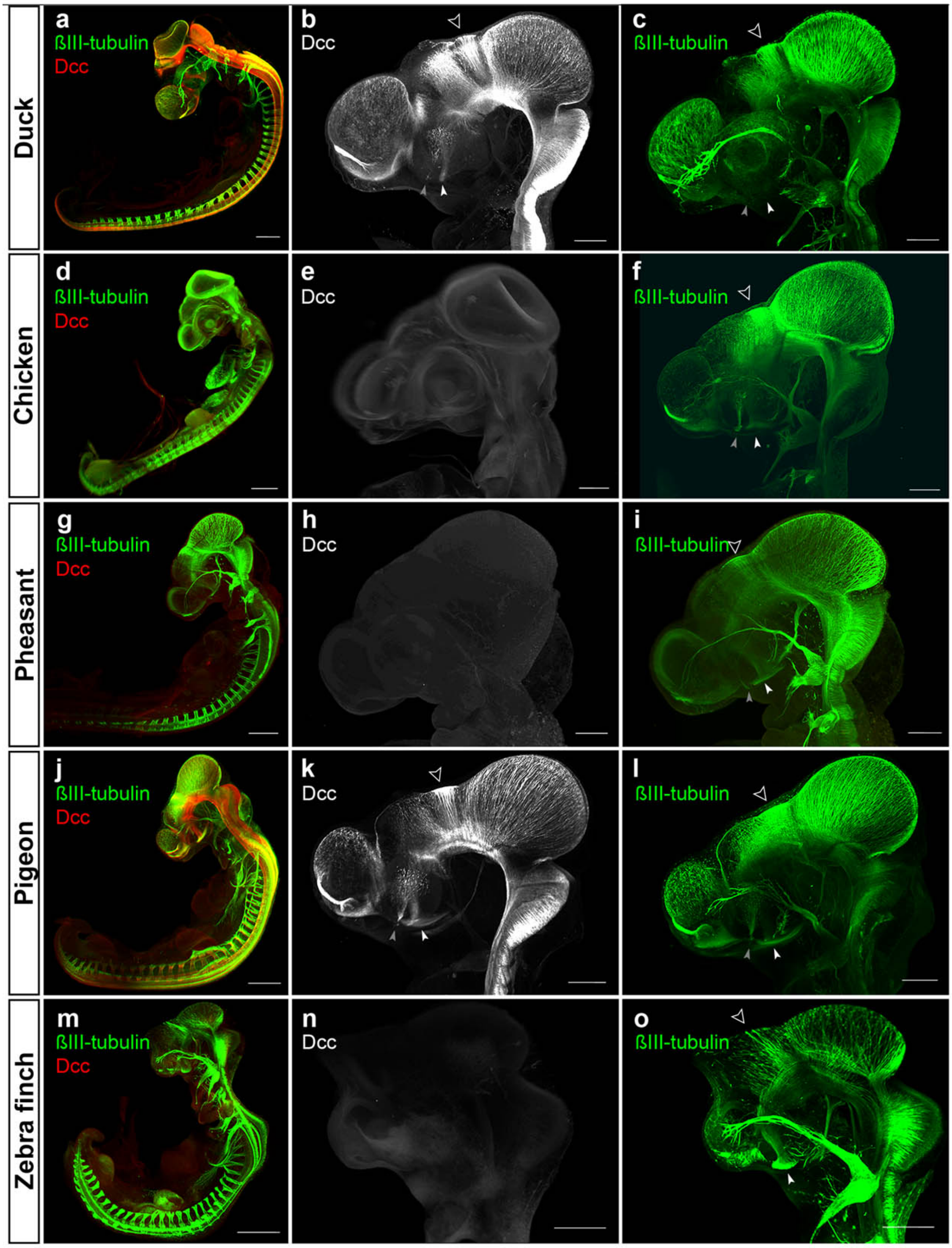

Figure S4. DCC expression in duck, chicken, pheasant, pigeon and zebra finch. Light sheet images of HH21-23 duck (**a-f-k**), chicken (**b-g-l**), pheasant (**c-h-m**), pigeon (**d-i-n**) and zebra finch (**e-j-o**) embryos after DCC and ßIII-tubulin whole-mount immunostaining and 3DISCO clearing. Open arrows indicate the posterior commissure, white arrowheads the post-optic commissure and grey arrowheads the optic nerve. Scale bars: 500µm (left); 200µm (right).

Table S1 – Names and references of DCC/NEOGENIN proteins identified and used in this study.

Clade		Order	Name	Latin name	Gene ID DCC	Gene ID Neogenin
			Spotted Gar	*Lepisosteus oculeatus*	ENSLOCP00000003949	XP_006629128.1
			Zebrafish	*Danio rerio*	NP_001030157.1	AAL92552.1
			Tilapia	*Oreochromis niloticus*	XP_005470639.1	XP_005455737.1
			Medaka	*Oryzias latipes*	XP_011480439.1	XP_011485992.1
			Tetraodon	*Tetraodon nigroviridis*	ENSTNIP00000017207	ENSTNIT00000012640
			Xenopus	*Xenopus tropicalis*	XP_004910423.1	ABA02169.1
			Opossum	*Monodelphis domestica*	ENSMODP00000013810	XP_007478277.1
			Elephant	*Loxodonta africana*	XP_003406765.1	XP_010592181.1
			Mouse	*Mus musculus*	ENSMUSP00000110593	AAH54540.1
			Human	*Homo sapiens*	ENSP00000389140	AAC51287.1
Sauropsid			Lizard	*Anolis carolinensis*	ENSACAP00000008535	XP_003227756.1
			Gekko	*Gekko japonicus*	XP_015283785.1	XP_015281163.1
			American Aligator	*Alligator mississippiensis*	XP_014449591.1	XP_014459957.1
			Chinese Alligator	*Alligator sinensis*	XP_006018932.1	XP_006026332.1
			Painted turtle	*Chrysemys picta bellii*	XP_005296926.1	XP_005281067.1
			Chinese turtle	*Pelodiscus sinensis*	XP_006136853.2	XP_006122251.2
			Sea Turtle	*Chelonia mydas*	XP_007063909.1	XP_007059969.1
	Aves	Paleo.	Ostrich	*Struthio camelus australis*	XP_009664396.0	XP_009673998.0
			Kiwi	*Apteryx australis mantelli*	XP_013800074.1	XP_013800108.1
			Tinamu	*Tinamus guttatus*	XP_010215865.1	XP_010223404.1
		Galli.	Chicken	*Gallus gallus*	ND	AAC59662.1
			Quail	*Coturnix Japonicus*	ND	XP_015727843.1
			Turkey	*Meleagris gallopovo*	ND	XP_003209386.1
		Ans.	Duck	*Anas platyrhynchos* (mallard)	XP_005011860.1	XP_005017275.1
			Goose	*Anser cygnoides domesticus*	XP_013050573.1	XP_013049547.1
		Striso.	Hummingbird	*Calypte anna*	ND	XP_008500653.1
			Swift	*Chaetura pelagica*	XP_010001093.1	XP_010004197.1
			chuck-will's-widow	*Caprimulgus carolinensis*	ND	XP_010166263.1
		Columbaves	Turaco	*Tauraco erythrolophus*	XP_009978908.1	XP_009991006.1
			Pigeon	*Columba livia*	XP_005503037.1	XP_005512248.1
			Sandgrouse	*Pterocles gutturalis*	ND	XP_010070753.1
			Cuckoo	*Cuculus canorus*	XP_009561651.1	XP_009567164.1
			Mesite	*Mesitornis unicolor*	Part. (XP_010181434.1)	Part. XP_010191234.1
		Gru.	Crowned crane	*Balearica regulorum gibbericeps*	XP_010310977.1	XP_010299714.1
			Bustard	*Chlamydotis macqueenii*	Part. (XP_010126392.1)	XP_010125600.1
		Aequorlitornithes	Loon	*Gavia stellata*	XP_009818994.1	XP_009811795.1
			Fulmar	*Fulmarus glacialis*	XP_009583160.1	XP_009579568.1
			Cormorant	*Phalacrocorax carbo*	ND	XP_009501723.1
			Pelican	*Pelecanus crispus*	XP_009476861.1	XP_009483113.1
			Egret	*Egretta grazetta*	XP_009635714.1	XP_009642196.1
			Ibis	*Nipponia nippon*	XP_009461288.1	XP_009467911.1
			Killdeer	*Charadrius vociferus*	XP_009884696.1	XP_009893858.1
			Ruff	*Calidris pugnax*	XP_014795236.1	XP_014809326.1
			Tropicbird	*Phaethon lepturus*	ND	XP_010290237.1
			Sunbittern	*Eurypyga helias*	ND	XP_010145839.1
			Adeli Penguin	*Pygoscelis adeliae*	XP_009321339.1	XP_009318182.1
			Emperor Penguin	*Aptenodytes forsteri*	XP_009273020.1	XP_009277190.1
		Acci.	Golden Eagle	*Aquila chrysaetos canadensis*	XP_011588980.1	XP_011588100.1
			White -tailed Eagle	*Haliaeetus albicilla*	XP_009927949.1	XP_009926840.1
			Bald Eagle	*Haliaeetus leucocephalus*	XP_010581336.1	XP_010569893.1
		Coracii.	Rhinoceros hornbill	*Buceros rhinoceros silvestris*	XP_010140526.1	XP_010144031.1
			Cuckoo roller	*Leptosomus discolor*	Part. (XP_009950574.1)	XP_009955259.1
			Bee-eater	*Merops nubicus*	ND	XP_008942215.1
			Woodpecker	*Picoides pubescens*	ND	XP_009904177.1
			Trogon	*Apaloderma vittatum*	Part. (XP_009875528.1)	Part.(XP_009863463.1)
			Hoatzin	*Opisthocomus hoazin*	XP_009943494.1	XP_009934163.1
			Seriema	*Cariama cristata*	ND	XP_009708043.1
		Falc.	Falcon	*Falco cherrug*	XP_005438019.1	XP_005437570.1
			Falcon	*Falco peregrinus*	XP_005242943.1	XP_005232662.1
		Psit.	Kea	*Nestor notabilis*	ND	XP_010011546.1
			Budgerigar	*Melopsittacus undulatus*	XP_012983556.1	XM_005145789.2
		Passeriforme	Ground tit	*Pseudopodoces humilis*	ND	XP_005521773.1
			Flycatcher	*Ficedula albicollis*	ND	XP_005051876.1
			Zebra Finch	*Taeniopygia guttata*	ND	XP_002190793.1
			Ground Finch	Geospiza fortis	ND	XP_005425990.1
			Sparrow	Zonotrichia albicollis	ND	XP_014124836.1
			Hooded crow	Corvus cornix	ND	XP_010410734.1
			Canary	Serinus canaria	ND	XP_009088347.1
			Manakin	Manacus vitellinus	ND	XP_008922371.1
			American crow	Corvus brachyrhynchos	ND	XP_008629530.1
			Starling	Sturnus vulgaris	ND	XP_014744736.1
			Rifleman	Acanthisitta chloris	ND	XP_009075656.1

Name	Latin name	Frazzled
Fruit fly	*Drosophila melanogaster*	NP_523716.2

Key :
- NCBI Genome Browser
- Ensembl Genome Browser
- Partial sequence
- ND : No Data

Paleo. Paleognathes
Galli. Galliformes
Ans. Anseriformes
Striso. Strisores
Gru. Gruiformes
Acci. Accipitriformes
Coracii. Coraciimorphes
Falc. Falconiformes
Psit. Psitaciformes

Table S2 – Names and references of NETRIN proteins identified and used in this study.

	Name	Latin name	Gene ID					
			NTN1 (a/b)		NTN2/3	NTN5	NTN4 (a/b)	
	Spotted Gar	*Lepisosteus oculeatus*	ENSLOCP00000016355		ENSLOCP00000010931	ENSLOCP00000017788	ENSLOCP00000019382	
	Zebrafish	*Danio rerio*	ENSDARP00000024821	ENSDARP00000038885	ENSDARP00000127895	ENSDARP00000052908	ENSDARP00000132076	
	Tilapia	*Oreochromis niloticus*	ENSONIP00000022872	ENSONIP00000010389	ENSONIP00000025223	ENSONIP00000023890	ENSONIP00000010787	ENSONIP00000022484
	Tetraodon	*Tetraodon nigroviridis*	ENSTNIP00000013104	ENSTNIP00000013104	ENSTNIP00000015771	ENSTNIP00000013058	ENSTNIP00000015647	ENSTNIP00000019104
	Xenopus	*Xenopus tropicalis*	ENSXETP00000042263		ENSXETP00000043788	ENSXETP00000063547	XP_002941301.2	
	Opossum	*Monodelphis domestica*	ENSMODP00000010543		ENSMODP00000019264	ENSMODP00000040382	ENSMODP00000010079	
	Elephant	*Loxodonta africana*	XP_003416830.1		ENSLAFP00000010538	ENSLAFP00000001628	ENSLAFP00000003437	
	Mouse	*Mus musculus*	ENSMUSP00000104314		NP_035077.1	ENSMUSP00000138412	ENSMUSP00000020204	
	Human	*Homo sapiens*	ENSP00000173229		ENSP00000293973	ENSP00000270235	ENSP00000340998	
Sauropsid	Lizard	*Anolis carolinensis*	XP_008102492.1		ENSACAP00000001541	ENSACAP00000015606	ENSACAP00000013843	
Sauropsid	Painted turtle	*Chrysemys picta bellii*	XP_005309923.1		ND	XP_005312122.1	XP_005306562.1	
Sauropsid	Sea Turtle	*Chelonia mydas*	XP_007072692.1		XP_007054595.1	XP_007067254.1	XP_007069305.1	
Sauropsid / Aves	Ostrich	*Struthio camelus australis*	XP_009672469.1		XP_009670387.1	ND	XP_009672888.1	
Sauropsid / Aves / Galli.	Chicken	*Gallus gallus*	ENSGALP00000038541		ENSGALP00000043056	ND	ENSGALP00000018590	
Sauropsid / Aves / Galli.	Turkey	*Meleagris gallopovo*	XP_010719536.1		XP_010718184.1	ND	XP_010707623.1	
Sauropsid / Aves	Falcon	*Falco peregrinus*	XP_005236273.1		XP_005241062.1	ND	XP_005242758.2	
Sauropsid / Aves	Ibis	*Nipponia nippon*	XP_009461473.1		XP_009460387.1	ND	XP_009462272.1	
Sauropsid / Aves	Pigeon	*Columba livia*	XP_005515376.2		XP_005505712.1	ND	XP_013225601.1	
Sauropsid / Aves / Pass.	Zebra Finch	*Taeniopygia guttata*	ENSTGUP00000005317		ENSTGUP00000003367	ND	ENSTGUP00000008736	
Sauropsid / Aves / Pass.	Ground Finch	*Geospiza fortis*	XP_005427923.1		XP_005425254.1	ND	XP_014162721.1	
Sauropsid / Aves / Pass.	Ground tit	*Pseudopodoces humilis*	XP_005531006.1		XP_005522839.1	ND	XP_014104309.1	

Name	Latin name	NTN1	NTN4
Amphioxus	*Branchiostoma floridae*	XP_002606106.1	XP_002209834.1

Key :

NCBI Genome Browser
Ensembl Genome Browser
Partial sequence
ND : No Data

Galli. Galliformes
Pass. Passeriformes

Table S3 – References and locations of the genes used in DCC synteny analysis.

	HDHD2			IER3IP1			SKOR2			SMAD2			ZBTB7C			CTIF		
Species	Chr.	scaffold	Begining..End	Chr.	scaffold	Begining..End	Chr.	scaffold	Begining..End	Chr.	scaffold	Begining..End	Chr.	scaffold	Begining..End	Chr.	scaffold	Begining..End
Human	18	NC_000018.10	47107410..47150515	18	NC_000018.10	47155019..47176374	18	NC_000018.10	47206203..47249630	18	NC_000018.10	47833095..47931193	18	NC_000018.10	48026864..48409422	18	NC_000018.10	48539056..48863217
Mouse	18	NC_000084.6	76944115..76972171	18	NC_000084.6	76930027..76941614	18	NC_000084.6	76846734..76901032	18	NC_000084.6	76241251..76311748	18	NC_000084.6	75820178..76148564	18	NC_000084.6	75431205..75697696
Platypus	3	NC_009096.1	30550731..30590997	3	NC_009096.1	30538527..30544956	3	NC_009096.1	30463258..30513782	3	NC_009096.1	30271255..30328445	Un	NW_001652091.1	3921..5130	3	NC_009096.1	21434957..21769483
Painted Turtle	6	NC_024223.1	21836464..21863053	6	NC_024223.1	21825557..21833432	6	NC_024223.1	21744018..21774562	6	NC_024223.1	21257039..21334983	6	NC_024223.1	20900288..21193172	6	NC_024223.1	20453990..20787469
Aligator S	Un	NW_005843543.1	51453..71041	Un	NW_005843543.1	76131..86890				Un	NW_005843076.1	200069..266615	Un	NW_005843076.1	33697..148259	Un	NW_005842412.1	850594..1175426
Duck	Un	NW_004677259.1	45073..57746	Un	NW_004677259.1	38924..42468	Un	NW_004685984.1	394..723	Un	NW_004677244.1	180986..196801	Un	NW_004676673.1	113721..194261	Un	NW_004678011.1	31036..58796
Goose	Un	NW_013185710.1	3909275..3927430	Un	NW_013185710.1	3930029..3932472	Un	NW_013185710.1	3951667..3979052	Un	NW_013185710.1	4196686..4241691	Un	NW_013185710.1	4268765..4307949	Un	NW_013185710.1	4445677..4571999
Chicken	Z	NC_006127.3	1757596..1767009	Z	NC_006127.3	1754071..1755692	Z	NC_006127.3	1720941..1743524	Z	NC_006127.3	1515759..1555720	Z	NC_006127.3	1429693..1495052	Z	NC_006127.3	1278212..1355316
Turkey	Z	NC_015041.2	1576283..1584504	Z	NC_015041.2	1571475..1574346	Z	NC_015041.2	1545081..1562277	Z	NC_015041.2	1368148..1374886	Z	NC_015041.2	1266738..1295584	Z	NC_015041.2	1124052..1214584
Quail	Z	NC_029547.1	1531593..1539216	Z	NC_029547.1	1530356..1531474	Z	NC_029547.1	1504058..1521362	Z	NC_029547.1	1314284..1352680	Z	NC_029547.1	1207623..1301142	Z	NC_029547.1	1116465..1192274
Falcon C	Un	NW_004994667.1	4157887..4173301				Un	NW_004994667.1	4104633..4156014	Un	NW_004994667.1	3832889..3874210	Un	NW_004994667.1	3783293..3790410	Un	NW_004994667.1	3447094..3561711
Bald Eagle	Un	NW_010973302.1	951055..966825	Un	NW_010973302.1	969206..973499				Un	NW_010973302.1	1247139..1299952	Un	NW_010973302.1	1330086..1337259	Un	NW_010973302.1	1553067..1663688
Adeli Penguin	Un	NW_008824669.1	791854..806461	Un	NW_008824669.1	784864..788039				Un	NW_008824669.1	457671..496337	Un	NW_008824669.1	407468..414268	Un	NW_008824669.1	63854..211692
Royal Eagle	Un	NW_011950951.1	960119..976432				Un	NW_011950951.1	978521..1030261	Un	NW_011950951.1	1256123..1309246	Un	NW_011950951.1	1339620..1346829	Un	NW_011950951.1	1568938..1684454
Pigeon	Un	NW_004973232.1	3268129..3283154	Un	NW_004973232.1	3286108..3288471	Un	NW_004973232.1	3309763..3334554	Un	NW_004973232.1	3583916..3627308	Un	NW_004973232.1	3662607..3669313	Un	NW_004973232.1/NW_004973534.1	3889068..3915888/17951..82570
Emperor Penguin	Un	NW_008795136.1	597730..614336	Un	NW_008795136.1	618203..623799	Un	NW_008795136.1	642422..668887	Un	NW_008795136.1	911437..951758	Un	NW_008795136.1	995556..1003598	Un	NW_008795136.1	1201450..1347225
Cuckoo	Un	NW_009243669.1	261662..270819	Un	NW_009243669.1	278839..290763	Un	NW_009243669.1	310012..338060	Un	NW_009245953.1	15758..58949	Un	NW_009245953.1	108777..116115	Un	NW_009245953.1	387342..51497
Ostrich	Un	NW_009270529.1	4501907..4516834	Un	NW_009270529.1	4495771..4498722				Un	NW_009270529.1	4174983..4226613	Un	NW_009270529.1	3993993..4146349	Un	NW_009270529.1	3687594..3895584
Egret	Un	NW_009270001.1	1965741..1980397	Un	NW_009270001.1	1946910..195553	Un	NW_009270001.1	1902597..1914105	Un	NW_009270001.1	1604152..1647590	Un	NW_009270001.1	1547744..1558435	Un	NW_009270001.1	1162140..1288075
Hoatzin	Un	NW_009902935.1	1704249..1718227	Un	NW_009902935.1	1689782..1698092	Un	NW_009902935.1	1645021..1671180	Un	NW_009902935.1	1360771..1403535	Un	NW_009902935.1	1306949..1314358	Un	NW_009902935.1	958122..1081234
Chimney Swift	Un	NW_009954546.1	63682..75361	Un	NW_009954546.1	50505..57929				Un	NW_009954404.1	60326..99062	Un	NW_009954404.1	13777..20797	Un	NW_009951939.1	172..118240
Ground Tit	Un	NW_005087597.1	1541764..1554895	Un	NW_005087597.1	1535419..1539567	Un	NW_005087597.1	1492852..1516833	Un	NW_005087597.1	1205683..1253084	Un	NW_005087597.1	1082092..1174564	Un	NW_005087597.1	831592..978977
Zebrafinch	Z	NC_011493.1	115826..127606	Z	NC_011493.1	132546..135970	Z	NC_011493.1	372314..375296	Z	NC_011493.1	405878..457467	Z	NC_011493.1	494572..572439	Z	NC_011493.1	744801..864770
Sparrow	Un	NW_005081629.1	1463354..1476727	Un	NW_005081629.1	1457388..1461579	Un	NW_005081629.1	1416263..1440789	Un	NW_005081629.1	1123678..1174306	Un	NW_005081629.1	942296..1095309	Un	NW_005081629.1	747580..893608
Flycatcher	Z	NC_021700.1	1563613..1577319	Z	NC_021700.1	1556789..1561881	Z	NC_021700.1	1508765..1539937	Z	NC_021700.1	1199178..1249620	Z	NC_021700.1	1089638..1165783	Z	NC_021700.1	804226..925897
Crow	Un	NW_008240031.1	2068376..2079754	Un	NW_008240031.1	2060180..2062455				Un	NW_008240031.1	1746059..1783700	Un	NW_008240031.1	1696784..1716647	Un	NW_008238323.1	829981..979265

	SMAD7			DYM			RPL17			LIPG			ACAA2		
Species	Chr.	scaffold	Begining..End	Chr.	scaffold	Begining..End	Chr.	scaffold	Begining..End	Chr.	scaffold	Begining..End	Chr.	scaffold	Begining..End
Human	18	NC_000018.10	48919853..48950711	18	NC_000018.10	49041474..49460709,	18	NC_000018.10	49488481..49492565	18	NC_000018.10	49560699..49592908	18	NC_000018.10	49783504..49813881
Mouse	18	NC_000084.6	75367365..75395935	18	NC_000084.6	75018699..75286966	18	NC_000084.6	74998978..75003381	18	NC_000084.6	74939322..74961263	18	NC_000084.6	74779212..74806207
Platypus	3	NC_009096.1	21860851..21873485	3	NC_009096.1	21963498..22225288	Un	NW_001640867.1	1425..7550	Un	NW_001697114.1	6375..26225	3	NC_009096.1	22337187..22360026
Painted Turtle	6	NC_024223.1	20305745..20349130	6	NC_024223.1	19865979..20204166	6	NC_024223.1	19839085..19847496	6	NC_024223.1	19790229..19806510	6	NC_024223.1	19586830..19610185
Aligator S	Un	NW_005842412.1	739088..783341	Un	NW_005842412.1	284762..658551	Un	NW_005842412.1	248810..254124	Un	NW_005842412.1	210282..221270	Un	NW_005842412.1	44944..67445
Duck	Un	NW_004678011.1	837..1767	Un	NW_004677464.1	44330..205978	Un	NW_004677464.1	210930..214155	Un	NW_004677464.1	220078..226253	Un	NW_004677170.1	391079..402424
Goose	Un	NW_013185710.1	4592341..4624062	Un	NW_013185710.1	4658581..4862440	Un	NW_013185710.1	4871403..4874459	Un	NW_013185710.1	4886966..4895141	Un	NW_013185710.1	4966218..4979707
Chicken	Z	NC_006127.3	1242360..1270480	Z	NC_006127.3	1086906..1234537	Z	NC_006127.3	1078176..1081526	Z	NC_006127.3	1062226..1069594	Z	NC_006127.3	985735..998075
Turkey	Un	NW_011200213.1	10369..12125	Z	NC_015041.2	1032078..1049047	Z	NC_015041.2	929317..932731	11	NC_015021.2	18865243..18873947	11	NC_015021.2	18936091..18948181
Quail	Z	NC_029547.1	1084374..1110488	Z	NC_029547.1	958582..1080688	Z	NC_029547.1	951619..954769	Z	NC_029547.1	935654..943946	Z	NC_029547.1	865968..877748
Falcon C	Un	NW_004994667.1	3391335..3418995	Un	NW_004994667.1	3134901..3349599	Un	NW_004994667.1	3122755..3126245	Un	NW_004994667.1	3099456..3109672	Un	NW_004994667.1	3004308..3056251
Bald Eagle	Un	NW_010973302.1	1690716..1716502	Un	NW_010973302.1	1756300..1975344	Un	NW_010973302.1	1984783..1988139	Un	NW_010973302.1	2002557..2011450	Un	NW_010973302.1	2090318..2108582
Adeli Penguin	Un	NW_008824669.1	12169..36963	Un	NW_008825081.1	152337..367937	Un	NW_008825081.1	128298..131523	Un	NW_008825081.1	104474..114476	Un	NW_008825081.1	1961..21566
Royal Eagle	Un	NW_011950951.1	1707425..1733113	Un	NW_011950951.1	1774621..1989253	Un	NW_011950951.1	2005352..2008716	Un	NW_011950951.1	2023157..2032078	Un	NW_011950951.1	2111533..2129626
Pigeon	Un	NW_004973534.1	106366..132498	Un	NW_004973534.1	172938..351938	Un	NW_004973534.1	369420..372732	Un	NW_004973534.1	385213..393745	Un	NW_004973534.1	463632..479236
Emperor Penguin	Un	NW_008795136.1	1378575..1403090	Un	NW_008794713.1	668099..887078	Un	NW_008794713.1	644700..649035	Un	NW_008794713.1	620885..630129	Un	NW_008794713.1	493468..517745
Cuckoo	Un	NW_009245953.1	544536..576603	Un	NW_009245953.1	627839..870753	Un	NW_009245953.1	899638..903803	Un	NW_009245953.1	924064..932338	Un	NW_009245953.1	1027368..1057170
Ostrich	Un	NW_009270529.1	3629394..3660375	Un	NW_009270529.1	3362416..3590537	Un	NW_009270529.1	3346709..3350267	Un	NW_009270529.1	3325107..3333808	Un	NW_009270529.1	3218492..3239041
Egret	Un	NW_009270001.1	1104829..1131942	Un	NW_009270001.1	817016..1053066	Un	NW_009270001.1	791785..795396	Un	NW_009270001.1	768756..778642	Un	NW_009270001.1	657902..679487
Hoatzin	Un	NW_009902935.1	924877..925755	Un	NW_009902935.1	618408..850885	Un	NW_009902935.1	593444..600074	Un	NW_009902935.1	570176..582772	Un	NW_009902935.1	459244..483339
Chimney Swift	Un	NW_009951939.1	159252..191123	Un	NW_009952361.1	20422..182386	Un	NW_009960015.1	140..331	Un	NW_009951787.1	14329..22048	Un	NW_009952442.1	467297..479415
Ground Tit	Un	NW_005087597.1	783416..810510	Un	NW_005087597.1	528760..739560	Un	NW_005087597.1	514749..519185	Un	NW_005087597.1	489366..499326	Un	NW_005087597.1	392201..409627
Zebrafinch	Z	NC_011493.1	890787..916686	Z	NC_011493.1	974880..1196389	Z	NC_011493.1	1212633..1217567	Z	NC_011493.1	1559660..1570385	Z	NC_011493.1	1446543..1464821
Sparrow	Un	NW_005081629.1	699557..725785	Un	NW_005081629.1	440285..654206	Un	NW_005081629.1	415904..420386	Un	NW_005081629.1	390678..400317	Un	NW_005081629.1	282666..300690
Flycatcher	Z	NC_021700.1	744138..775342	Z	NC_021700.1	469764..698843	Z	NC_021700.1	455307..459579	Z	NC_021700.1	427337..437512	Z	NC_021700.1	318599..340895
Crow	Un	NW_008238323.1	770450..795684	Un	NW_008238323.1	526304..726235	Un	NW_008238323.1	501454..506378	Un	NW_008238323.1	477168..485992	Un	NW_008238323.1	380091..394790

	MYO5b			CFAP53 - (CCDC11)			SKA1			MAPK4			ME2			ELAC1		
Species	Chr.	scaffold	Begining..End	Chr.	scaffold	Begining..End	Chr.	scaffold	Begining..End	Chr.	scaffold	Begining..End	Chr.	scaffold	Begining..End	Chr.	scaffold	Begining..End
Human	18	NC_000018.10	49822786..50195081	18	NC_000018.10	50227193..50266495	18	NC_000018.10	50375022..50394168	18	NC_000018.10	50560114..50731826	18	NC_000018.10	50878604..50949795	18	NC_000018.10	50968017..50988120
Mouse	18	NC_000084.6	74442378..74772698	18	NC_000084.6	74283100..74359984	18	NC_000084.6	74195299..74207818	18	NC_000084.6	73928486..74065559	18	NC_000084.6	73764956..73815478	18	NC_000084.6	73735038..73754758
Platypus	3	NC_009096.1	22376877..22574525	3	NC_009096.1	22719179..22750151	3	NC_009096.1	22780706..22797744	3	NC_009096.1	22954866..23014792	3	NC_009096.1	23154045..23197780	3	NC_009096.1	23203814..23215851
Painted Turtle	6	NC_024223.1	19344675..19561566	6	NC_024223.1	19133491..19163849	6	NC_024223.1	19005904..19048880	6	NC_024223.1	18797963..18944617	6	NC_024223.1	18724168..18765281	6	NC_024223.1	18698169..18712209
Aligator S	Un	NW_005842412.1/NW_005842609.1	8479..35472/4626..252151	Un	NW_005842609.1	391939..425809	Un	NW_005842609.1	485330..526036	Un	NW_005842609.1	626972..758082	Un	NW_005842609.1	788667..833986	Un	NW_005842609.1	842319..849201
Duck	Un	NW_004677170.1	320417..389878	Un	NW_004677170.1	309337..318280	Un	NW_004677170.1	290397..298399	Un	NW_004677170.1	277861..282521	Un	NW_004677170.1	261742..271651	Un	NW_004677170.1	255878..258997
Goose	Un	NW_013185710.1	4979907..5059434	Un	NW_013185710.1	5074297..5086070	Un	NW_013185710.1	5102591..5113577	Un	NW_013185710.1	5142986..5155357	Un	NW_013185710.1	5166679..5185174	Un	NW_013185710.1	5188677..5194347
Chicken	28	NC_006115.3	1127762..1139008				Un	NW_003770383.1	671..1323									
Turkey	30	NC_015040.2	1085812..1096802				Un	NW_011126173.1	366..1587									
Quail	28	NC_029543.1	604749..615683				Un	NW_015439969.1	13231..20024				Un	NW_015439872.1	26752..49750			
Falcon C	Un	NW_004994667.1	2876360..2997990	Un	NW_004994667.1	2820559..2842067	Un	NW_004994667.1	2738554..2794390	Un	NW_004994667.1	2714622..2737972	Un	NW_004994667.1	2663567..2697987	Un	NW_004994667.1	2655680..2660591
Bald Eagle	Un	NW_010973302.1	2114978..2237931	Un	NW_010973302.1	2270518..2288025	Un	NW_010973302.1	2316068..2331648	Un	NW_010973302.1	2374312..2396060	Un	NW_010973302.1	2413167..2449759	Un	NW_010973302.1	2452022..2455142
Adeli Penguin	Un	NW_008824676.1	2879334..3001075	Un	NW_008824676.1	2819390..2841791	Un	NW_008824676.1	2778689..2794094	Un	NW_008824676.1	2711442..2734868	Un	NW_008824676.1	2660772..2687775	Un	NW_008824676.1	2652311..2656878
Royal Eagle	Un	NW_011950951.1	2131562..2286611	Un	NW_011950951.1	2292859..2311182	Un	NW_011950951.1	2340863..2358532	Un	NW_011950951.1	2378724..2425010	Un	NW_011950951.1	2438243..2475076	Un	NW_011950951.1	2475592..2480794
Pigeon	Un	NW_004973534.1	481210..529832	Un	NW_004973860.1	5585..17744	Un	NW_004974021.1	1743..11700	Un	NW_004973280.1	21431..31303	Un	NW_004973280.1	37546..55481	Un	NW_004973280.1	57915..61973
Emperor Penguin	Un	NW_008794713.1	358031..493002	Un	NW_008794713.1	279317..319905	Un	NW_008794713.1	256119..271565	Un	NW_008794713.1	188331..212805	Un	NW_008794713.1	140495..173197	Un	NW_008794713.1	129975..134872
Cuckoo	Un	NW_009245953.1	1057626..1226122	Un	NW_009245953.1	1236452..1261338				Un	NW_009245391.1	36437..63243	Un	NW_009245391.1	95998..129465	Un	NW_009245391.1	143308..147393
Ostrich	Un	NW_009270529.1	3105638..3213928	Un	NW_009270529.1	3055064..3073390	Un	NW_009270529.1	3004197..3025105	Un	NW_009270529.1	2898822..2963896	Un	NW_009270529.1	2842541..2878931	Un	NW_009270529.1	2830465..2837178
Egret	Un	NW_009270001.1	514179..648922	Un	NW_009270001.1	460320..479268	Un	NW_009270001.1	409414..428092	Un	NW_009270001.1	336415..359216	Un	NW_009270001.1	282992..309084	Un	NW_009270001.1	272289..275307
Hoatzin	Un	NW_009902935.1	323881..457735	Un	NW_009902935.1	257824..290540	Un	NW_009902935.1	224999..240658	Un	NW_009902935.1	155740..179311	Un	NW_009902935.1	103145..128525	Un	NW_009902935.1	93161..96633
Chimney Swift	Un	NW_009952442.1	325665..457687	Un	NW_009952442.1	308150..322752	Un	NW_009952442.1	266473..282851	Un	NW_009952442.1	210273..229436	Un	NW_009952442.1	159510..190153	Un	NW_009952442.1	145876..149679
Ground Tit	Un	NW_005087597.1	247583..386851	Un	NW_005087597.1	225829..241884	Un	NW_005087597.1	180937..196970	Un	NW_005087597.1	116244..136648	Un	NW_005087597.1	67890..97506	Un	NW_005087597.1	56647..62632
Zebrafinch	Z	NC_011493.1	1283315..1439797	Z	NC_011493.1	1261058..1277110	Z	NC_011493.1	1591016..1602384	Un	NW_002206047.1	6755..18001	Un	NW_002206047.1	42763..80753	Un	NW_002206047.1	84166..86850
Sparrow	Un	NW_005081629.1	157868..277144	Un	NW_005081629.1	105868..124358	Un	NW_005081629.1	58311..72740	Un	NW_005083733.1	2308..10863	Un	NW_005082072.1	46392..60029			
Flycatcher	Z	NC_021700.1	151510..313078)	Z	NC_021700.1	118293..146180	Z	NC_021700.1	62320..77644	Un	NW_004775956.1	90002..134087	Un	NW_004775956.1	18284..58588	Un	NW_004775956.1	15635..18648
Crow	Un	NW_008238323.1	256694..370920				Un	NW_008238323.1	158184..172399	Un	NW_008238323.1	95033..119291	Un	NW_008238323.1	42472..68101	Un	NW_008238323.1	34173..37331

	SMAD4			MEX3C			DCC			MBD2			POLi		
Species	Chr.	scaffold	Begining..End	Chr.	scaffold	Begining..End	Chr.	scaffold	Begining..End	Chr.	scaffold	Begining..End	Chr.	scaffold	Begining..End
Human	18	NC_000018.10	51030213..51085042	18	NC_000018.10	51174550..51197681	18	NC_000018.10	52340172..53535903	18	NC_000018.10	54151601..54224788	18	NC_000018.10	54269479..54321266
Mouse	18	NC_000084.6	73638998..73703741	18	NC_000084.6	73572585..73592578	18	NC_000084.6	71253613..72351231	18	NC_000084.6	70568270..70626131	18	NC_000084.6	70508680..70530334
Platypus	3	NC_009096.1	23243725..23267033	3	NC_009096.1	23328408..23360084	3	NC_009096.1	24402915..25070419	3	NC_009096.1	25408193..25657790	3	NC_009096.1	25467526..25507822
Painted Turtle	6	NC_024223.1	18648739..18682215	6	NC_024223.1	18565921..18598493	6	NC_024223.1	17067846..18041247	6	NC_024223.1	16671301..16732259	6	NC_024223.1	16643121..16660998
Aligator S	Un	NW_005842609.1	873969..896966	Un	NW_005842609.1	956281..959511	Un	NW_005841915.1	921977..1594571	Un	NW_005841915.1	1980420..2036850	Un	NW_005841915.1	2042387..2055952
Duck	Un	NW_004677170.1	238982..251653	Un	NW_004677170.1	232272..235066	Un	NW_004677170.1/NW_004676479.1	83..49858/22081..196467	Un	NW_004676479.1	278663..297813	Un	NW_004676479.1	310366..324070
Goose	Un	NW_013185710.1	5203409..5215395	Un	NW_013185710.1	5223447..5234971	Un	NW_013185836.1	33180..367673	Un	NW_013185836.1	501342..525170	Un	NW_013185836.1	526643..549691
Chicken										Un	NT_467280.1	5509..12391			
Turkey															
Quail	Un	NW_015439872.1	58422..112716							Un	NW_015439872.1	127509..154210			
Falcon C	Un	NW_004994667.1	2624085..2642037	Un	NW_004994667.1	2609574..2614320	Un	NW_004994667.1	1811388..2364694	Un	NW_004994667.1	1630024..1657836	Un	NW_004994667.1	1604516..1614433
Bald Eagle	Un	NW_010973302.1	2472497..2488881	Un	NW_010973302.1	2496862..2514015	Un	NW_010973302.1	2737176..3307683	Un	NW_010973302.1	3472060..3513924	Un	NW_010973302.1	3514797..3525174
Adeli Penguin	Un	NW_008824676.1	2616477..2635426	Un	NW_008824676.1	2583987..2605886	Un	NW_008824676.1	1780684..2360396	Un	NW_008824676.1	1573991..1604229	Un	NW_008824676.1	1547486..1561676
Royal Eagle	Un	NW_011950951.1	2497711..2514123	Un	NW_011950951.1	2522400..2567488	Un	NW_011950951.1	2766973..3338870	Un	NW_011950951.1	3511084..3554115	Un	NW_011950951.1	3554372..3565178
Pigeon	Un	NW_004973280.1	69249..82443	Un	NW_004973280.1	87642..93042	Un	NW_004973280.1	293376..501989	Un	NW_004973280.1	585719..600651	Un	NW_004973280.1	605432..610907
Emperor Penguin	Un	NW_008794713.1	99175..113535	Un	NW_008794713.1	82368..84152	Un	NW_008794883.1	2535474..3125515	Un	NW_008794883.1	2331135..2361592	Un	NW_008794883.1	2304975..2318688
Cuckoo	Un	NW_009245391.1	169063..187176	Un	NW_009245391.1	205823..227775	Un	NW_009245391.1	525953..693291	Un	NW_009245391.1	700907..705832	Un	NW_009245391.1	711041..719168
Ostrich	Un	NW_009270529.1	2789725..2809216	Un	NW_009270529.1	2778195..2785522	Un	NW_009270529.1	1914301..2524592	Un	NW_009270529.1	1676361..1706541	Un	NW_009270529.1	1648439..1661197
Egret	Un	NW_009270001.1	240891..255099	Un	NW_009270001.1	165015..225172	Un	NW_009259496.1	1806587..2178367	Un	NW_009259496.1	1594021..1631885	Un	NW_009259496.1	1582138..1592405
Hoatzin	Un	NW_009902935.1	62378..76146	Un	NW_009902935.1	46292..47655	Un	NW_009900148.1	10545..221925	Un	NW_009900148.1	388276..421424	Un	NW_009900148.1	434543..443263
Chimney Swift	Un	NW_009952442.1	113871..127417	Un	NW_009952442.1	104696..105921	Un	NW_009952042.1	707123..867976	Un	NW_009952042.1	622301..636009	Un	NW_009952042.1	598239..607025
Ground Tit	Un	NW_005087597.1	22200..48306	Un	NW_005087597.1	8925..14893									
Zebrafinch	Un	NW_002224671.1	NW_002224671.1												
Sparrow															
Flycatcher															
Crow	Un	NW_008238323.1	7696..18285	Un	NW_008239113.1	76037..78375									

	STARD6			DYNAP			RAB27b			CCDC68			TCF4			TXNL1		
Species	Chr.	scaffold	Begining..End	Chr.	scaffold	Begining..End	Chr.	scaffold	Begining..End	Chr.	scaffold	Begining..End	Chr.	scaffold	Begining..End	Chr.	scaffold	Begining..End
Human	18	NC_000018.10	54324592..54357890	18	NC_000018.10	54532713..54599493	18	NC_000018.10	54717866..54895516	18	NC_000018.10	54901509..54959508	18	NC_000018.10	55222331..55635993	18	NC_000018.10	56602387..56638689,
Mouse	18	NC_000084.6	70472454..70505254	18	NC_000084.6	70240429..70244584	18	NC_000084.6	69979131..70141633	18	NC_000084.6	69924858..69969484	18	NC_000084.6	69344490..69687967	18	NC_000084.6	63662801..63692359
Platypus	3	NC_009096.1	25507883..25530728	3	NC_009096.1	25680774..25686338	3	NC_009096.1	25762767..25864981	3	NC_009096.1	25875360..25889021	3	NC_009096.1	26163873..26583829	3	NC_009096.1	27265310..27301660
Painted Turtle				6	NC_024223.1	16584186..16608461	6	NC_024223.1	16349859..16544153	6	NC_024223.1	16316830..16339584	6	NC_024223.1	15863408..16183662	6	NC_024223.1	15299469..15324159
Aligator S	Un	NW_005841915.1	2055892..2072999	Un	NW_005841915.1	2106158..2114286	Un	NW_005841915.1	2348560..2408132	Un	NW_005841915.1	2417087..2449359	Un	NW_005841915.1	2607468..2967937	Un	NW_005841915.1	3527404..3556026
Duck	Un	NW_004676479.1	317748..324480	Un	NW_004676479.1	332388..342204	Un	NW_004676479.1	343027..367264	Un	NW_004676479.1	372295..375699	Un	NW_004676479.1	416094..583231	Un	NW_004677656.1	26382..40183
Goose	Un	NW_013185836.1	545068..552731	Un	NW_013185836.1	561770..573037)	Un	NW_013185836.1	573979..618974	Un	NW_013185836.1	620029..625556	Un	NW_013185836.1	675746..881809	Un	NW_013185836.1	1095661..1112172
Chicken													Z	NC_006127.3	875..141070	Z	NC_006127.3	593098..607664
Turkey										Un	NW_011210716.1	203..4666				Z	NC_015041.2	508369..523641
Quail													Z	NC_029547.1	14672..73045	Z	NC_029547.1	525041..539307
Falcon C	Un	NW_004994667.1	1597525..1604430	Un	NW_004994667.1	1565651..1572888	Un	NW_004994667.1	1503066..1512237				Un	NW_004994667.1	1195882..1429427	Un	NW_004994667.1	928482..946469
Bald Eagle	Un	NW_010973302.1	3516353..3533706	Un	NW_010973302.1	3552282..3561973	Un	NW_010973302.1	3620116..3629252				Un	NW_010973302.1	3710307..3949691	Un	NW_010973302.1	4201718..422738
Adeli Penguin	Un	NW_008824676.1	1539955..1546975	Un	NW_008824676.1	1500102..1519259	Un	NW_008824676.1	1439395..1448138	Un	NW_008824676.1	1424773..1429472	Un	NW_008824676.1	1108603..1353834	Un	NW_008824676.1	836862..858419
Royal Eagle	Un	NW_011950951.1	3555631..3573943	Un	NW_011950951.1	3589462..3602994	Un	NW_011950951.1	3667814..3677587	Un	NW_011950951.1	3690025..3690510	Un	NW_011950951.1	3753829..4001131	Un	NW_011950951.1	4253363..4279161
Pigeon	Un	NW_004973280.1	611072..617468	Un	NW_004973280.1	630398..636251	Un	NW_004973280.1	659633..663628	Un	NW_004973280.1	668243..672147	Un	NW_004973280.1	721514..888567	Un	NW_004973280.1	1028515..104921
Emperor Penguin	Un	NW_008794883.1	2297340..2304455	Un	NW_008794883.1	2269145..2275905	Un	NW_008794883.1	2195524..2204390				Un	NW_008794883.1	1859356..2111599	Un	NW_008794883.1	1569450..1602095
Cuckoo													Un	NW_009245391.1	745757..836282	Un	NW_009245391.1	854660..858373
Ostrich	Un	NW_009270529.1	1641389..1648332	Un	NW_009270529.1	1613248..1620631	Un	NW_009270529.1	1527716..1580315	Un	NW_009270529.1	1502192..1522034	Un	NW_009270529.1	1199073..1426830	Un	NW_009270529.1	920067..938245
Egret	Un	NW_009259496.1	1574596..1580982				Un	NW_009259496.1	1484350..1490987	Un	NW_009259496.1	1468296..1472523	Un	NW_009259496.1	1143971..1396872	Un	NW_009259496.1	845446..869002
Hoatzin	Un	NW_009900148.1	444630..451021	Un	NW_009900148.1	466627..479931	Un	NW_009900148.1	533822..544576				Un	NW_009900148.1	625231..887836	Un	NW_009900148.1	1135818..1155794
Chimney Swift	Un	NW_009952042.1	592284..597249				Un	NW_009952042.1	518365..530250	Un	NW_009952042.1	508938..516423	Un	NW_009952042.1	223638..455206	Un	NW_009952042.1	15340..27661
Ground Tit													Un	NW_005087834.1	2402..97593	Un	NW_005087924.1	26829..37574
Zebrafinch																Z	NW_002234477.1	37108..47593
Sparrow																Un	NW_005082890.1	171..11768
Flycatcher													Un	NW_004775940.1	61678..65112	Un	NW_004775940.1	148830..163014
Crow													Un	NW_008239118.1	10100..140306	Un	NW_008239118.1	252978..260726

	WDR7			ST8SIA3			FECH			NARS			ATB8B1		
Species	Chr.	scaffold	Begining..End	Chr.	scaffold	Begining..End	Chr.	scaffold	Begining	Chr.	scaffold	Begining..End	Chr.	scaffold	Begining..End
Human	18	NC_000018.10	56651343..57029805	18	NC_000018.10	57352490..57371800	18	Ensembl	57544841..57586737	18	NC_000018.10	57600656..57621945	18	NC_000018.10	57646426..57803822
Mouse	18	NC_000084.6	63708695..63989760	18	NC_000084.6	64157724..64277180	18	NC_000084.6	64456542..64489095	18	NC_000084.6	64499647..64516557	18	NC_000084.6	64528979..64661860
Platypus	3	NC_009096.1	27368281..27786298	3	NC_009096.1	28033376..28048599	3	NC_009096.1	28234360..28262777	3	NC_009096.1	28277937..28294510	3	NC_009096.1	28304854..28429520
Painted Turtle	6	NC_024223.1	14913466..15265891	6	NC_024223.1	14733961..14739852	6	NC_024223.1	14580904..14608881	6	NC_024223.1	14526741..14552051	6	NC_024223.1	14454469..14508523
Aligator S	Un	NW_005841915.1	3561196..3889561	Un	NW_005841922.1	124025..128558	Un	NW_005841922.1	251320..285416	Un	NW_005841922.1	290141..310661	Un	NW_005841922.1	326541..368344
Duck	Un	NW_004677656.1/NW_004680303.1	665..20930/3607..61175	Un	NW_004680303.1	101916..106751	Un	NW_004680303.1	151325..163908	Un	NW_004680303.1	164042..175070	Un	NW_004680303.1	176739..191152
Goose	Un	NW_013185836.1	1118140..1225055	Un	NW_013185836.1	1273542..1285062	Un	NW_013185836.1	1333825..1348099	Un	NW_013185836.1	1351600..1363328	Un	NW_013185836.1	1365256..1398124
Chicken	Z	NC_006127.3	477166..592930	Z	NC_006127.3	435849..439872	Z	NC_006127.3	380954..391800	Z	NC_006127.3	367004..377724	Z	NC_006127.3	342471..365812
Turkey	Z	NC_015041.2	394076..508186	Z	NC_015041.2	351533..37035	Z	NC_015041.2	200433..209130	Z	NC_015041.2	210012..216198	Z	NC_015041.2	226124..237905
Quail	Z	NC_029547.1	417990..524840	Z	NC_029547.1	371575..380844	Z	NC_029547.1	323677..337102	Z	NC_029547.1	311862..322136	Z	NC_029547.1	273785..306029
Falcon C	Un	NW_004994667.1	785974..913583	Un	NW_004994667.1	720883..737352	Un	NW_004994667.1	633942..664046	Un	NW_004994667.1	622819..633342	Un	NW_004994667.1	595016..620920
Bald Eagle	Un	NW_010973302.1	4237038..4370939	Un	NW_010973302.1	4405853..4434001	Un	NW_010973302.1	4483817..4510463	Un	NW_010973302.1	4517987..4533172	Un	NW_010973302.1	4534713..4562967
Adeli Penguin	Un	NW_008824676.1	688317..822213	Un	NW_008824676.1	620050..626518	Un	NW_008824676.1	534555..556795	Un	NW_008824676.1	513887..527418	Un	NW_008824676.1	480092..510499
Royal Eagle	Un	NW_011950951.1	4276546..4424815	Un	NW_011950951.1	4427961..4494042	Un	NW_011950951.1	4544588..4566528	Un	NW_011950951.1	4574062..4589084	Un	NW_011950951.1	4591055..4619359
Pigeon	Un	NW_004973280.1	1049300..1103206	Un	NW_004973280.1	1106220..1142112	Un	NW_004973280.1	1176731..1191946	Un	NW_004973280.1	1197561..1207121	Un	NW_004973280.1	1207327..1224259
Emperor Penguin	Un	NW_008794883.1	1424210..1559180	Un	NW_008794883.1	1351905..1397814	Un	NW_008794883.1	1267428..1290203	Un	NW_008794883.1	1245315..1260332	Un	NW_008794883.1	1214647..1243369
Cuckoo	Un	NW_009245391.1	860971..872928	Un	NW_009245391.1	873347..877222	Un	NW_009245391.1	909334..923180	Un	NW_009245391.1	924628..935396	Un	NW_009245391.1	1317355..1347574
Ostrich	Un	NW_009270529.1	742387..915886	Un	NW_009270529.1	638120..648038	Un	NW_009270529.1	540188..565502	Un	NW_009270529.1	512113..529318	Un	NW_009270529.1	479115..509537
Egret	Un	NW_009259496.1	705666..836198	Un	NW_009259496.1	643256..657474	Un	NW_009259496.1	559361..572879	Un	NW_009259496.1	537810..553059	Un	NW_009259496.1	511586..537390
Hoatzin	Un	NW_009900148.1	1170606..1289356	Un	NW_009901407.1	46111..49844	Un	NW_009900945.1	585492..598463	Un	NW_009900945.1	566131..578287	Un	NW_009900945.1	540110..565397
Chimney Swift	Un	NW_009954235.1	616554..705174	Un	NW_009954235.1	563140..612071	Un	NW_009954235.1	483896..498884	Un	NW_009954235.1	467191..482274	Un	NW_009954235.1	441300..466162
Ground Tit	Un	NW_005087924.1/NW_005087720.1	443..26120/406076..424216	Un	NW_005087720.1	371491..375768	Un	NW_005087720.1	324047..339146	Un	NW_005087720.1	312837..319891	Un	NW_005087720.1	293453..311529
Zebrafinch	Z	NW_002234477.1	48474..86318	Z	NW_002234477.1	110750..116374	Z	NW_002234477.1	159702..169891	Z	NW_002234477.1	170930..180537	Z	NW_002234477.1	181527..199944
Sparrow	Un	NW_005082890.1/NW_005081874.1	12748..19044/8..26179	Un	NW_005081874.1	59386..65350	Un	NW_005081874.1	94992..104590	Un	NW_005081874.1	110428..117726	Un	NW_005081874.1	120052..139660
Flycatcher	Un	NW_004775940.1	167693..204972	Un	NW_004775940.1	235139..241661	Un	NW_004775940.1	282620..296690	Un	NW_004775940.1	297266..304171	Un	NW_004775940.1	305757..328897
Crow	Un	NW_008239118.1	266175..304868	Un	NW_008239118.1	332298..336997	Un	NW_008239118.1	370967..379826	Un	NW_008239118.1	382545..390450	Un	NW_008239118.1	391260..403980

	NEDD4L			ALPK2			MALT1			Znf532			SEC11C		
Species	Chr.	scaffold	Begining..End	Chr.	scaffold	Begining..End	Chr.	scaffold	Begining..End	Chr.	scaffold	Begining..End	Chr.	scaffold	Begining..End
Human	18	NC_000018.10	58044233..58401541	18	NC_000018.10	58481247..58629188	18	NC_000018.10	58671386..58750139	18	NC_000018.10	58862600..58986477	18	NC_000018.10	59139884..59158837
Mouse	18	NC_000084.6	64887756..65217826	18	NC_000084.6	65265529..65393888,	18	NC_000084.6	65430969..65478888	18	NC_000084.6	65580230..65689443	18	NC_000084.6	65800499..65817660
Platypus	3	NC_009096.1	28713350..28811546	3	NC_009096.1	28937546..29062247	3	NC_009096.1	29094388..29188573	3	NC_009096.1	29269574..29351216	3	NC_009096.1	29432724..29437420
Painted Turtle	Un	NW_007359882.1	111400..469642	Un	NW_007359882.1	548679..621672	Un	NW_007359882.1	659289..731542	Un	NW_007359882.1	802184..885474	Un	NW_007359882.1	990083..1002594
Aligator S	Un	NW_005841922.1	622304..894794	Un	NW_005841922.1	997059..1062637	Un	NW_005841922.1	1093316..1162342	Un	NW_005841922.1	1276377..1329383	Un	NW_005841922.1	1428173..1441394
Duck	Un	NW_004677402.1	83650..188103	Un	NW_004677402.1	187557..257808	Un	NW_004677402.1	255671..284190	Un	NW_004677402.1	306897..352369	Un	NW_004677402.1	367085..371815
Goose	Un	NW_013185836.1	1444987..1582854	Un	NW_013185836.1	1580356..1663374	Un	NW_013185836.1	1652940..1694109	Un	NW_013185836.1	1714634..1759246	Un	NW_013185836.1	1773859..1779475
Chicken	Z	NC_006127.3	613201..742966	Z	NC_006127.3	768082..807313	Z	NC_006127.3	808518..841792	Z	NC_006127.3	853144..892040	Z	NC_006127.3	899405..904533
Turkey	Z	NC_015041.2	663872..713578	Z	NC_015041.2	764939..798193	Z	NC_015041.2	807232..836019	Z	NC_015041.2	849331..889605	Z	NC_015041.2	897815..902967
Quail	Z	NC_029547.1	544702..659646	Z	NC_029547.1	678714..715758	Z	NC_029547.1	716965..745510	Z	NC_029547.1	753955..791720	Z	NC_029547.1	793168..797733
Falcon C	Un	NW_004994667.1	356558..410616	Un	NW_004994667.1	278105..313108	Un	NW_004994667.1	234366..278095	Un	NW_004994667.1	157923..192791	Un	NW_004994667.1	130477..136310
Bald Eagle	Un	NW_010973302.1	4662993..4809678	Un	NW_010973302.1	4853113..4892838	Un	NW_010973302.1	4905815..4943173	Un	NW_010973302.1	4973616..5023329	Un	NW_010973302.1	5043945..505126
Adeli Penguin	Un	NW_008824676.1	215211..366409	Un	NW_008824676.1	127453..176428	Un	NW_008824676.1	88891..127433	Un	NW_008824676.1	3233..46976	Un	NW_008825324.1	17191..27217
Royal Eagle	Un	NW_011950951.1	4723002..4876325	Un	NW_011950951.1	4912539..4964839	Un	NW_011950951.1	4967138..5008274	Un	NW_011950951.1	5036336..5087927	Un	NW_011950951.1	5107329..5114132
Pigeon	Un	NW_004973280.1/NW_004974495.1	1284629..1355457/46..4525	Un	NW_004974495.1	4575..54561	Un	NW_004974495.1	66338..87631	Un	NW_004974495.1	92040..124172	Un	NW_004974495.1	145181..149989
Emperor Penguin	Un	NW_008794883.1	939772..1107804	Un	NW_008794883.1	854933..902275	Un	NW_008794883.1	812755..854913	Un	NW_008794883.1	727168..776202	Un	NW_008794442.1	138540..148540
Cuckoo	Un	NW_009245391.1	1164144..1242463	Un	NW_009245391.1	1104782..1127332	Un	NW_009245391.1	1064396..1102968	Un	NW_009245391.1	1015070..1042552	Un	NW_009245391.1	998828..1003531
Ostrich	Un	NW_009270529.1	186493..399340	Un	NW_009270529.1	85561..143171	Un	NW_009270529.1	37738..84254	Un	NW_009270033.1	149733..203000	Un	NW_009270033.1	124383..130259
Egret	Un	NW_009259496.1	301679..415041	Un	NW_009259496.1	231797..264871	Un	NW_009259496.1	187908..225503	Un	NW_009259496.1	122039..155965	Un	NW_009259496.1	102161..107551
Hoatzin	Un	NW_009900945.1	314070..447642	Un	NW_009900945.1	240743..274747	Un	NW_009900945.1	197328..233758	Un	NW_009900945.1	126825..162028	Un	NW_009900945.1	103419..108841
Chimney Swift	Un	NW_009954235.1	257479..357864	Un	NW_009954235.1	187908..219732	Un	NW_009954235.1	155610..187909	Un	NW_009954235.1	98465..124255	Un	NW_009954235.1	78270..82783
Ground Tit	Un	NW_005087720.1	185612..276078	Un	NW_005087720.1	135687..179579	Un	NW_005087720.1	114939..134299	Un	NW_005087720.1	70353..102634	Un	NW_005087720.1	57772..62927
Zebrafinch	Z	NW_002234477.1	245437..307242	Z	NW_002234477.1	329338..346697	Z	NW_002234477.1	357539..376098	Z	NW_002234477.1	387542..422032	Z	NW_002234477.1	427942..433267
Sparrow	Un	NW_005081874.1	175861..218719	Un	NW_005081874.1	238465..262138	Un	NW_005081874.1	264132..279864	Un	NW_005081874.1	291163..319660	Un	NW_005081874.1	326350..330578
Flycatcher	Un	NW_004775940.1	361571..429882	Un	NW_004775959.1	156719..167833	Un	NW_004775959.1	120676..137974	Un	NW_004775959.1	76167..109493	Un	NW_004775959.1	63521..7100
Crow	Un	NW_008239118.1	458074..533246	Un	NW_008239118.1	568491..586878	Un	NW_008239118.1	591013..613304	Un	NW_008239118.1	628218..658820	Un	NW_008239118.1	666753..670759

	GRP			RAX			CPLX4			LMAN1		
Species	Chr.	scaffold	Begining..End	Chr.	scaffold	Begining..End	Chr.	scaffold	Begining..End	Chr.	scaffold	Begining..End
Human	18	NC_000018.10	59219118..59230771	18	NC_000018.10	59267035..59273393	18	NC_000018.10	59295402..59318649	18	NC_000018.10	59327823..59359276
Mouse	18	NC_000084.6	65872748..65886599	18	NC_000084.6	65934639..65939089	18	NC_000084.6	65955722..65970178	18	NC_000084.6	65980739..66002635
Platypus	3	NC_009096.1	29443187..29466040	3	NC_009096.1	29498105..29502812	3	NC_009096.1	29521386..29558452	3	NC_009096.1	29563877..29582203
Painted Turtle	Un	NW_007359882.1	1031319..1039992	Un	NW_007359882.1	1063027..1065685	Un	NW_007359882.1	1093245..1121956	Un	NW_007359882.1	1140783..1173783
Aligator S	Un	NW_005841922.1	1462918..1477182	Un	NW_005841922.1	1495458..1497066	Un	NW_005841922.1	1521339..1545335	Un	NW_005841922.1	1559863..1585429
Duck	Un	NW_004677402.1	383671..387259	Un	NW_004677402.1	394117..395840	Un	NW_004677402.1	404990..425594	Un	NW_004677402.1	427008..445630
Goose	Un	NW_013185836.1	1791092..1798064	Un	NW_013185836.1	1804045..180721	Un	NW_013185836.1	1816617..1833927	Un	NW_013185836.1	1838421..1857249
Chicken	Z	NC_006127.3	911940..916603	Z	NC_006127.3	921298..923007	Z	NC_006127.3	928029..941837	Z	NC_006127.3	942874..962370,
Turkey	Z	NC_015041.2	911397..915299				11	NC_015021.2	18995337..19011229	11	NC_015021.2	18976079..18992794
Quail	Z	NC_029547.1	804682..808637	Z	NC_029547.1	812509..814155,	Z	NC_029547.1	818887..832396	Z	NC_029547.1	832450..850429
Falcon C	Un	NW_004994667.1	113068..118234	Un	NW_004994667.1	101730..104927	Un	NW_004994667.1	78062..94899	Un	NW_004994667.1	50067..70359
Bald Eagle	Un	NW_010973302.1	5061051..5069816	Un	NW_010973302.1	5078771..507987	Un	NW_010973302.1	5090526..5107755	Un	NW_010973302.1	5115375..5140415
Adeli Penguin	Un	NW_008825324.1	33544..43880	Un	NW_008825324.1	53074..53571	Un	NW_008825324.1	66192..84826	Un	NW_008825324.1	88886..113503
Royal Eagle	Un	NW_011950951.1	5123860..5134103	Un	NW_011950951.1	5142514..5146101	Un	NW_011950951.1	5155310..5173570	Un	NW_011950951.1	5180752..5203308
Pigeon	Un	NW_004974495.1	152147..159246	Un	NW_004974495.1	215515..216953	Un	NW_004974495.1	198821..208782	Un	NW_004974495.1	164990..196563
Emperor Penguin	Un	NW_008794442.1	122028..132243	Un	NW_008794442.1	106540..112692	Un	NW_008794442.1	79764..98716	Un	NW_008794442.1	7773..76254
Cuckoo	Un	NW_009245391.1	986876..991906	Un	NW_009245391.1	980423..980737	Un	NW_009245391.1	963477..972908	Un	NW_009245391.1	940402..956386
Ostrich	Un	NW_009270033.1	109244..113172				Un	NW_009270033.1	66415..85387	Un	NW_009270033.1	24879..62907
Egret	Un	NW_009259496.1	83766..97343	Un	NW_009259496.1	75196..75654	Un	NW_009259496.1	53310..65651	Un	NW_009259496.1	17272..49102
Hoatzin	Un	NW_009900945.1	89331..93184				Un	NW_009900945.1	61425..72864	Un	NW_009900945.1	35317..55132
Chimney Swift	Un	NW_009954235.1	67845..76794	Un	NW_009954235.1	52658..59856	Un	NW_009954235.1	39236..51685	Un	NW_009954235.1	19084..33645
Ground Tit	Un	NW_005087720.1	50666..54875	Un	NW_005087720.1	43966..46415	Un	NW_005087720.1	28288..36038	Un	NW_005087720.1	12392..25640
Zebrafinch	Z	NW_002234477.1	436092..440339	Z	NW_002234477.1	444749..446681	Z	NW_002234477.1	453703..461648	3	NW_002198531.1	1159..10307
Sparrow	Un	NW_005081874.1	334259..338076				Un	NW_005081874.1	353642..360595	Un	NW_005081874.1	363185..376596
Flycatcher	Un	NW_004775959.1	58067..62829	Un	NW_004775959.1	50681..52132	Un	NW_004775959.1	34247..42086	Un	NW_004775959.1	17746..29555
Crow				Un	NW_008239118.1	683722..691316	Un	NW_008239118.1	693022..701323	Un	NW_008239118.1	706495..743481

Table S4 – Names of the genes used in DCC synteny analysis.

Short name	Full name
**HDHD2**	haloacid dehalogenase-like hydrolase domain containing 2
**IER3IP1**	immediate early response 3 interacting protein 1
**SKOR2**	SKI family transcriptional corepressor 2
**SMAD2**	SMAD family member 2
**ZBTB7C**	zinc finger and BTB domain containing 7C
**CTIF**	CBP80/20-dependent translation initiation factor
**SMAD7**	SMAD family member 7
**DYM**	dymeclin
**RPL17**	60S ribosomal protein L17
**LIPG**	lipase, endothelial
**ACAA2**	acetyl-CoA acyltransferase 2
**MyoVb**	myosin VB
**CFAP53**	cilia and flagella associated protein 53
**SKA1**	spindle and kinetochore associated complex subunit 1
**MAPK4**	mitogen-activated protein kinase 4
**ME2**	malic enzyme 2, NAD(+)-dependent, mitochondrial
**ELAC1**	elaC ribonuclease Z 1
**SMAD4**	SMAD family member 4
**MEX3C**	mex-3 RNA binding family member C
**DCC**	Deleted in Colorectal Carcinoma
**MBD2**	methyl-CpG binding domain protein 2
**POLi**	polymerase (DNA directed) iota
**STARD6**	StAR-related lipid transfer (START) domain containing 6
**DYNAP**	dynactin associated protein
**RAB27B**	RAB27B, member RAS oncogene family
**CCDC68**	coiled-coil domain containing 68
**TCF4**	transcription factor 4
**TXNL1**	thioredoxin-like 1
**WDR7**	WD repeat domain 7
**ST8SIA3**	ST8 alpha-N-acetyl-neuraminide alpha-2,8-sialyltransferase 3
**FECH**	ferrochelatase
**NARS**	asparaginyl-tRNA synthetase
**ATP8B1**	ATPase, aminophospholipid transporter, class I, type 8B, member 1
**NEDD4L**	neural precursor cell expressed, developmentally downregulated 4-like
**ALPK2**	alpha-kinase 2
**MALT1**	mucosa associated lymphoid tissue lymphoma translocation gene 1
**ZNF532**	zinc finger protein 532
**SEC11C**	SEC11 homologue C (S. cerevisiae)
**GRP**	gastrin-releasing peptide
**RAX**	retina and anterior neural fold homeobox
**CPLX4**	complexin 4
**LMAN1**	lectin, mannose-binding, 1

Table S5 – List of species used in DCC synteny analysis.

Species	Latin name
Human	*Homo sapiens*
Mouse	*Mus musculus*
Platypus	*Ornithorhynchus anatinus*
Painted Turtle	*Chrysemys picta bellii*
Alligator S	*Alligator sinensis*
Duck	*Anas platyrhynchos* (mallard)
Goose	*Anser cygnoides domesticus*
Chicken	*Gallus gallus*
Turkey	*Meleagris gallopavo*
Quail	*Coturnix japonica*
Saker Falcon	*Falco cherrug*
Bald Eagle	*Haliaeetus leucocephalus*
Adeli Penguin	*Pygoscelis adeliae*
Royal Eagle	*Aquila chrysaetos canadensis*
Pigeon	*Columba livia*
Emperor Penguin	*Aptenodytes forsteri*
Ibis	*Nipponia nippon*
Cuckoo	*Cuculus canorus*
Ostrich	*Struthio camelus australis*
Egret	*Egretta garzetta*
Hoatzin	*Opisthocomus hoazin*
Chimney Swift	*Chaetura pelagica*
Tibetan ground Tit	*Pseudopodoces humilis*
Zebra finch	*Taeniopygia guttata*
Sparrow	*Zonotrichia albicollis*
Flycatcher	*Ficedula albicollis*
Crow	*Corvus brachyrhynchos*

Table S6 – Recapitulation of animal samples used in this study: names, stages and staining.

Species	Incubation time (days)	Hamburger-Hamilton stage or equivalent	Total number
Chicken	4	HH21-22	8
	6	HH27-28	8
	8	HH31	5
	10	HH36	3
Duck	4,5	21	5
	6	23	4
	8	27-28	7
	10	30	3
Pigeon	4	21	2
	7	25	1
	9	30	3
Zebra Finch	4	22	6
	7	31	3
Quail	4	22	2
Pheasant	4	21	3
Partridge	4	21	3

Movie S1

Triple whole-mount immunolabeling for DCC (blue), ßIII-Tubulin (yellow) and Doublecortin (red), followed by 3Disco clearing in embryos from 4 bird species (Duck, Chicken, Pigeon and Zebra finch).

Movie S2

ROBO3 whole-mount immunolabeling and 3Disco clearing in a HH22 chick embryo.

Movie S3

ROBO3 whole-mount immunolabeling and 3Disco clearing in a 4 day-old pigeon embryo.
